# Supplementary figures and images for: Journal article publishing in the social sciences and humanities: A comparison of Web of Science coverage for five European countries
Source: PLoS One. 2021 Apr 8;16(4):e0249879. doi: 10.1371/journal.pone.0249879 (PMC8031415; doi:10.1371/journal.pone.0249879)

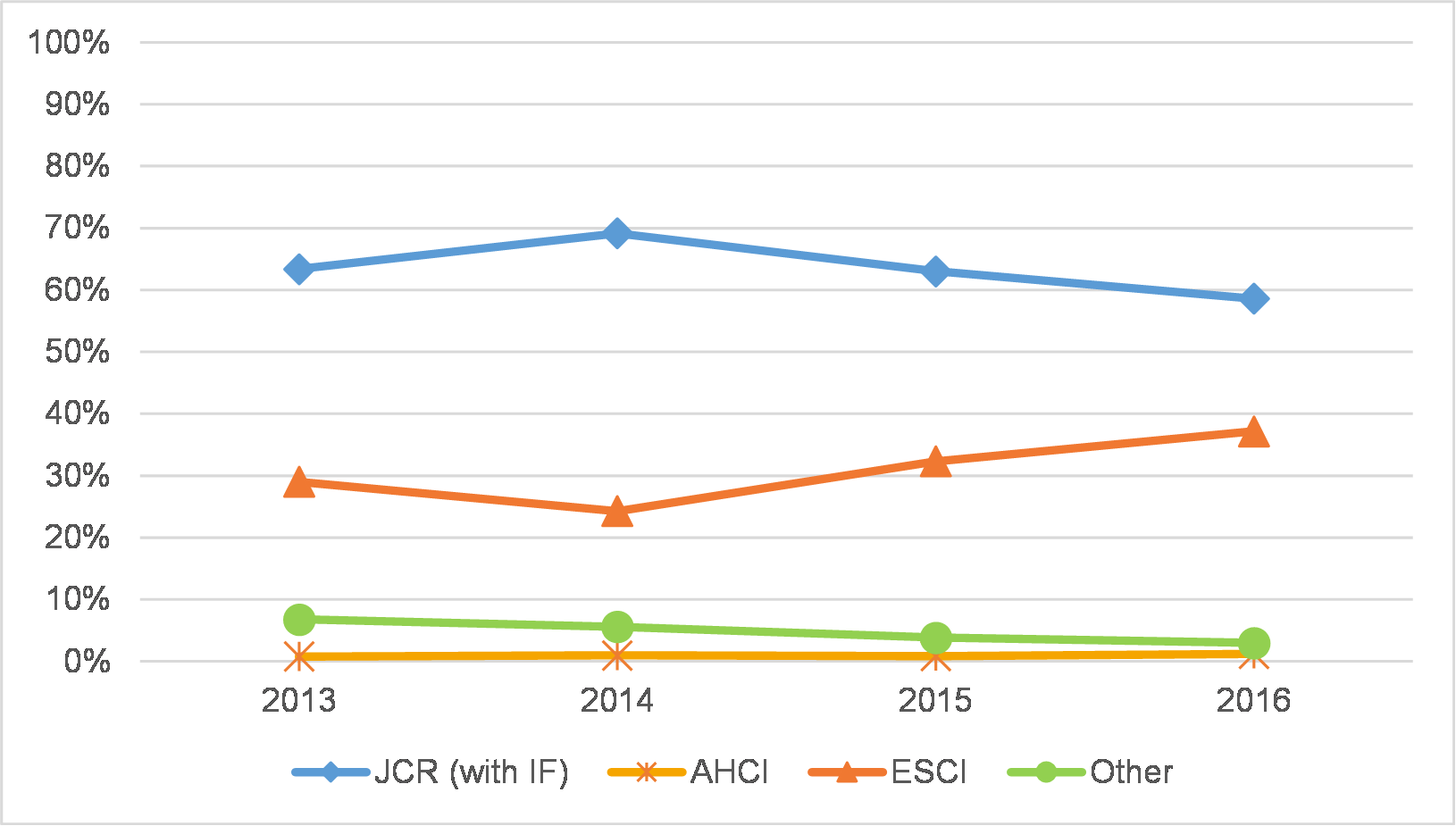

Supplement: S1 Fig — Czech Republic–social sciences. (TIF) [file pone.0249879.s001.tif]

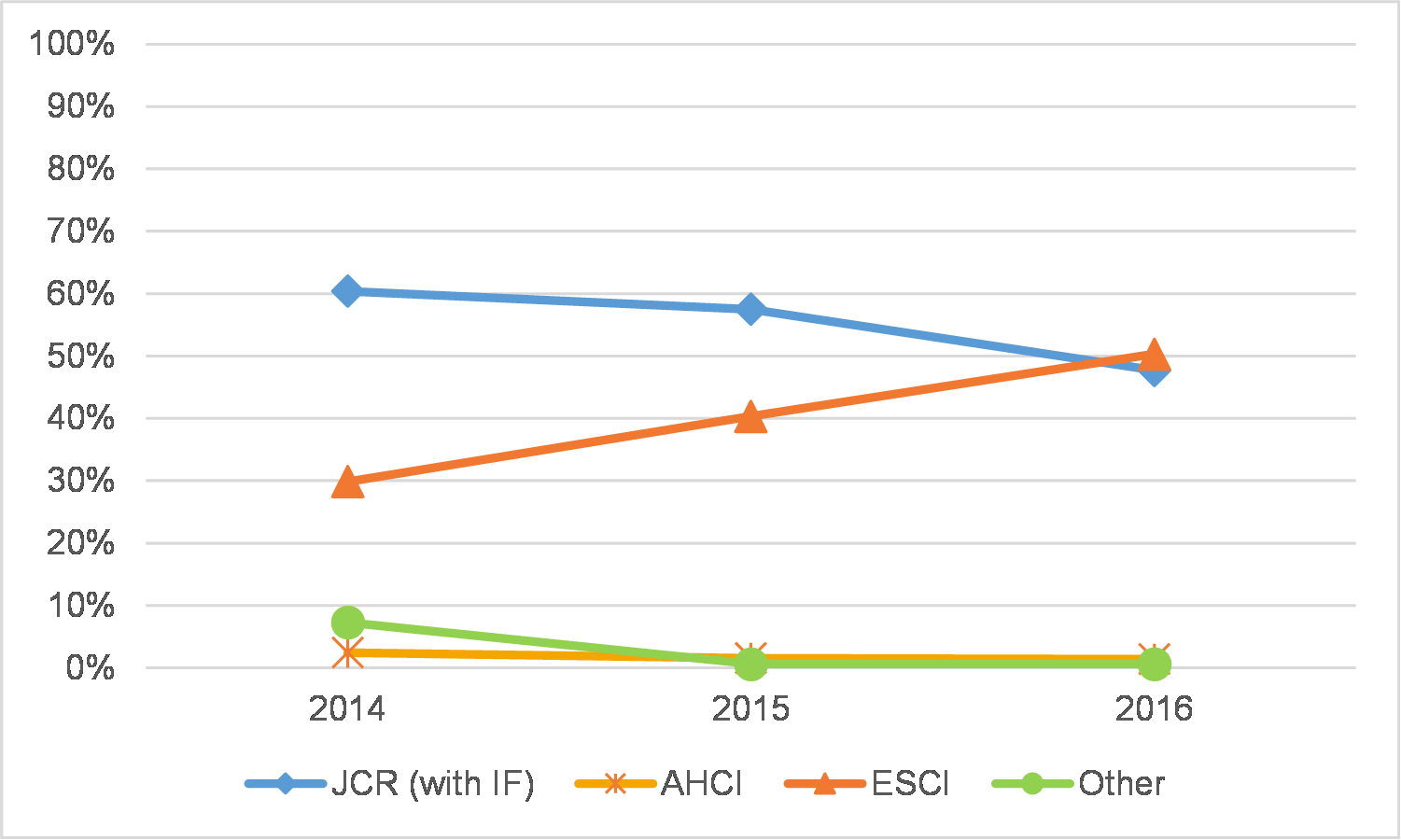

Supplement: S2 Fig — Slovakia–social sciences. (TIF) [file pone.0249879.s002.tif]

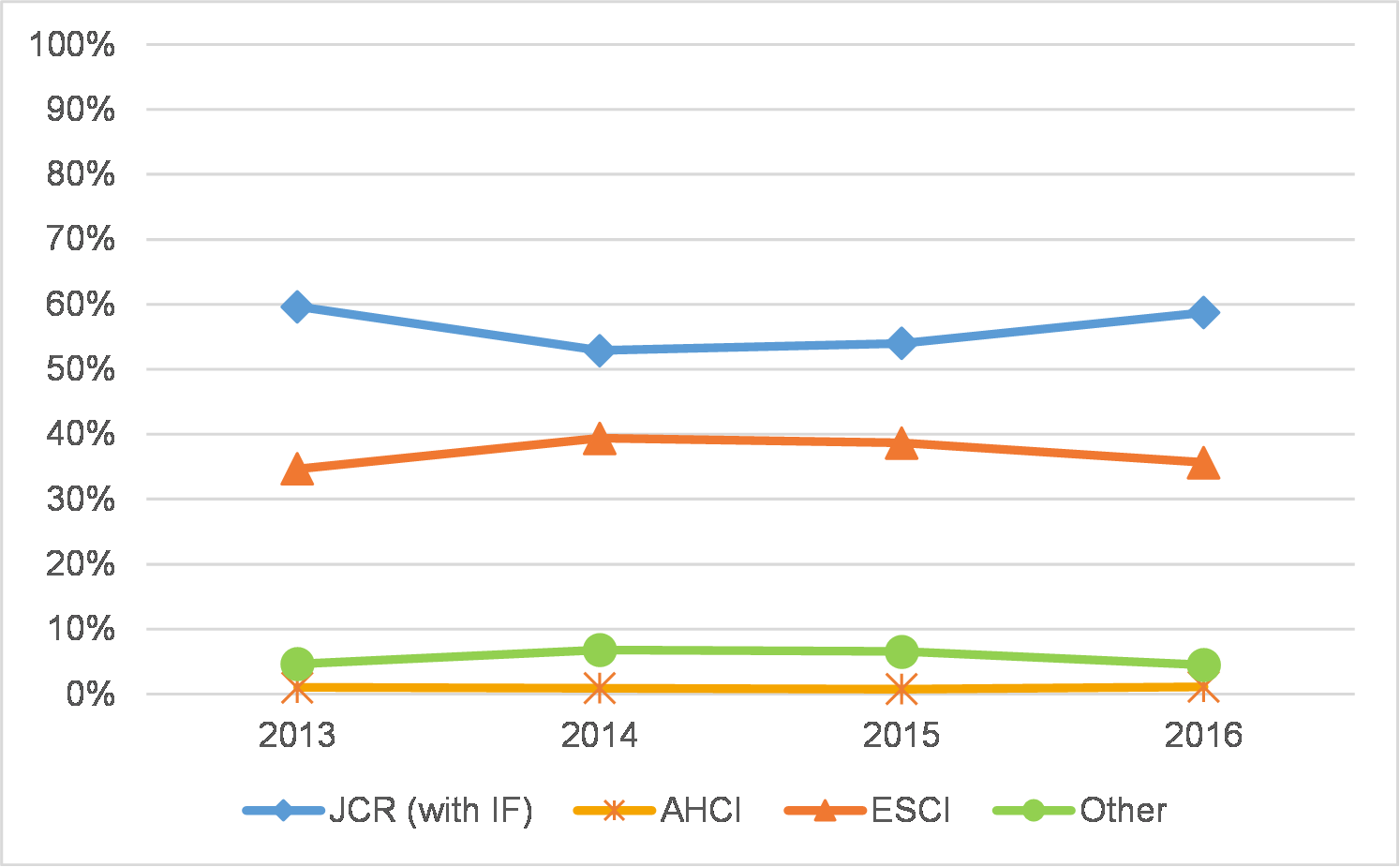

Supplement: S3 Fig — Poland–social sciences. (TIF) [file pone.0249879.s003.tif]

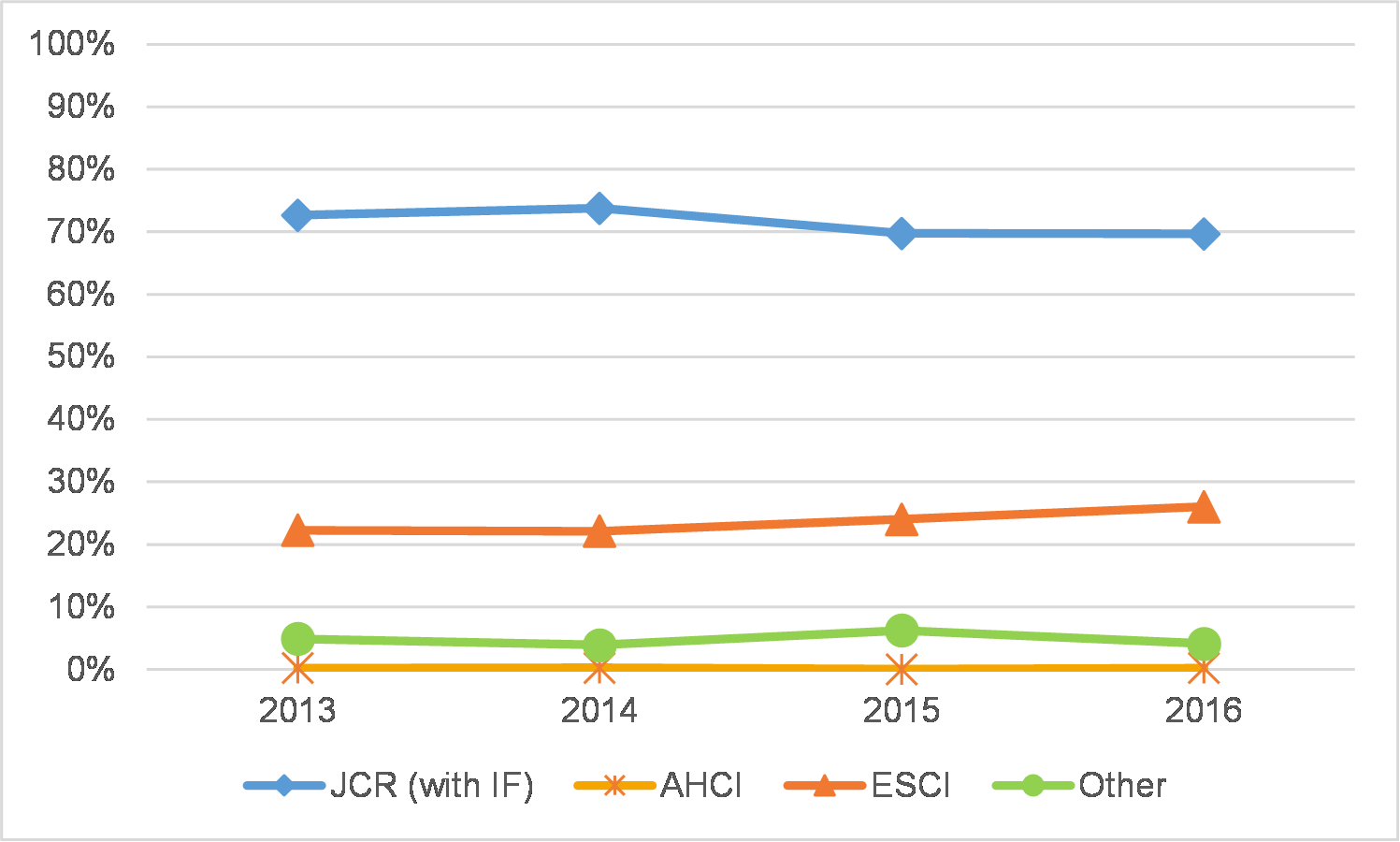

Supplement: S4 Fig — Norway–social sciences. (TIF) [file pone.0249879.s004.tif]

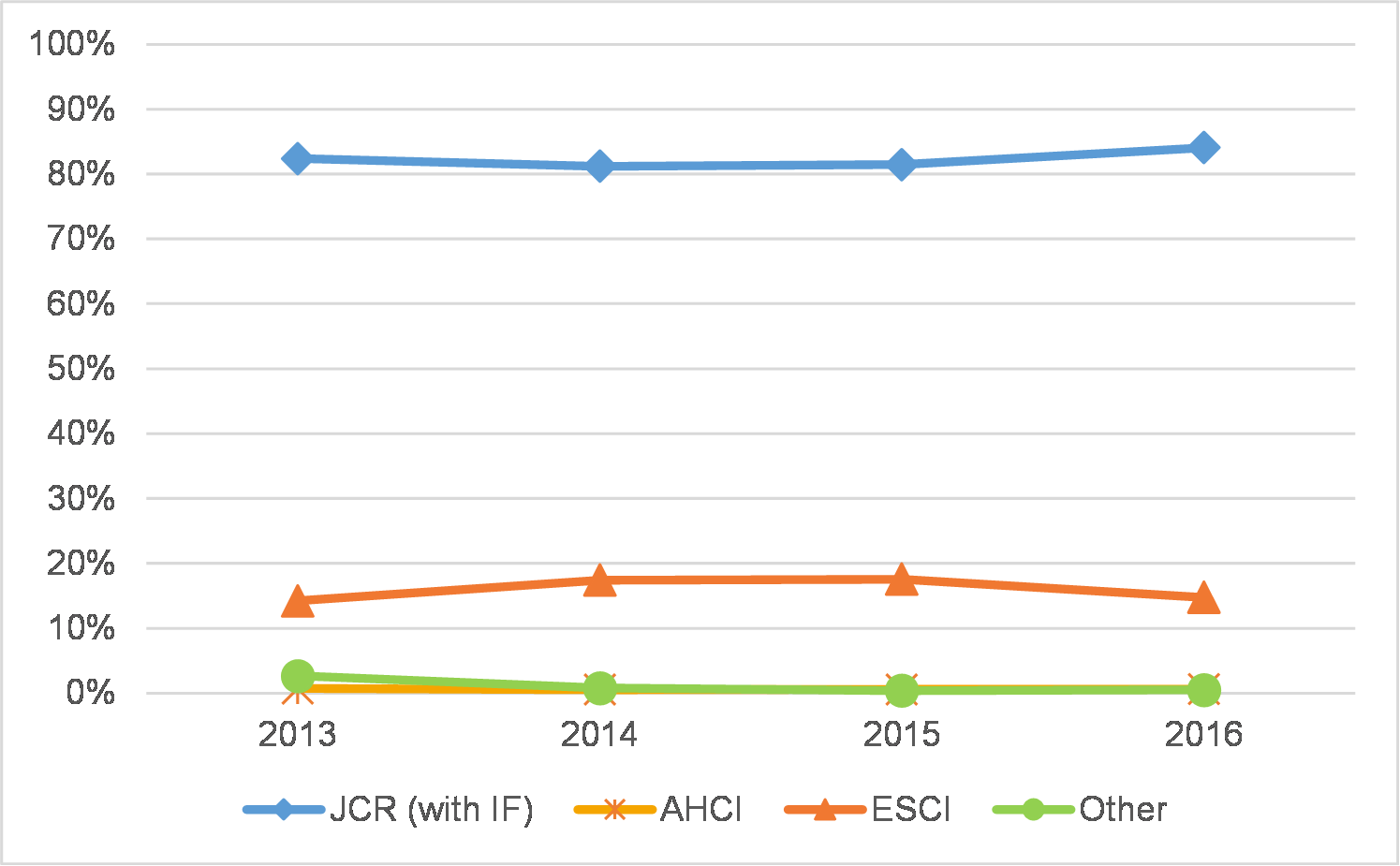

Supplement: S5 Fig — Flanders–social sciences. (TIF) [file pone.0249879.s005.tif]

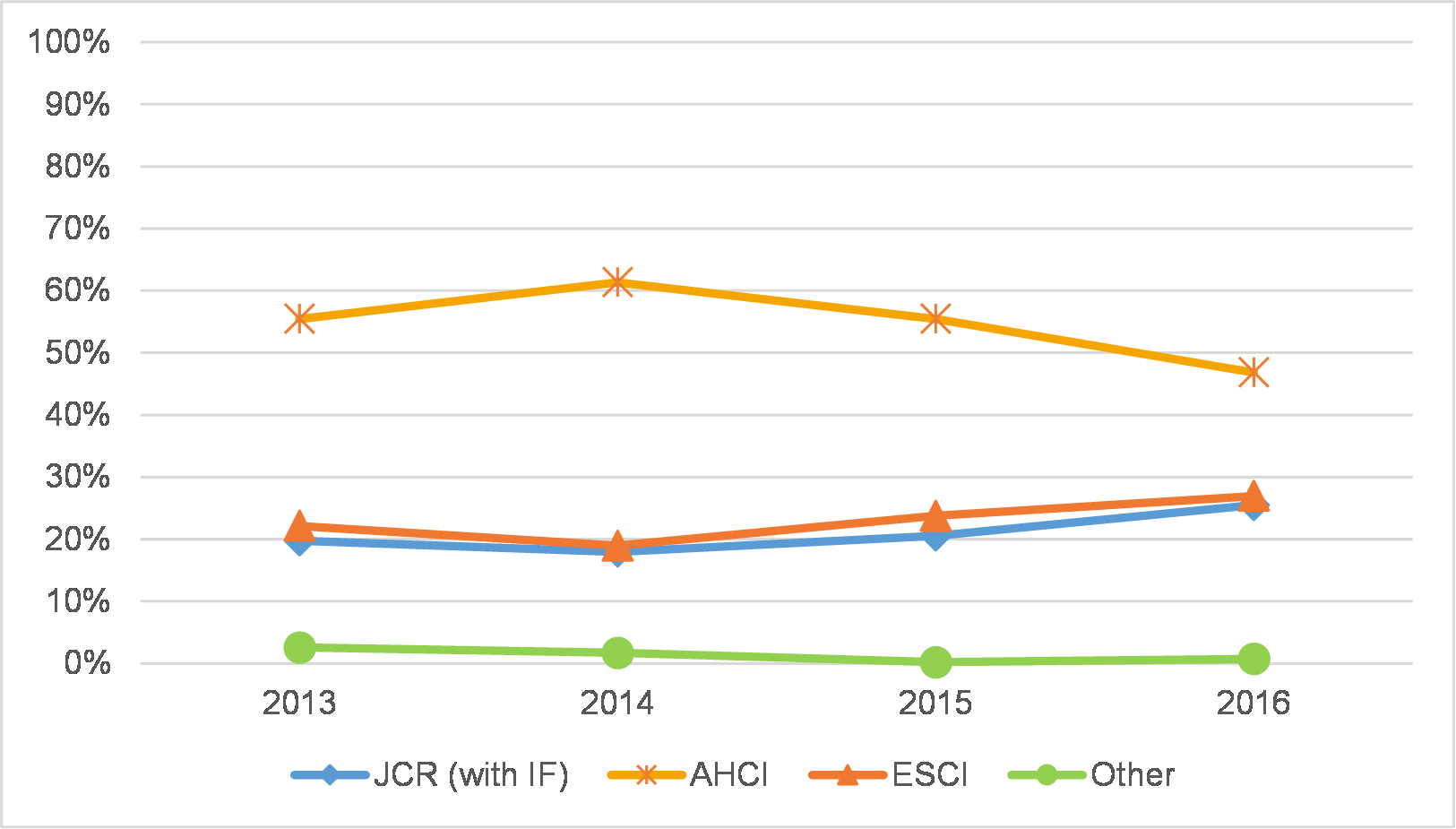

Supplement: S6 Fig — Czech Republic–humanities. (TIF) [file pone.0249879.s006.tif]

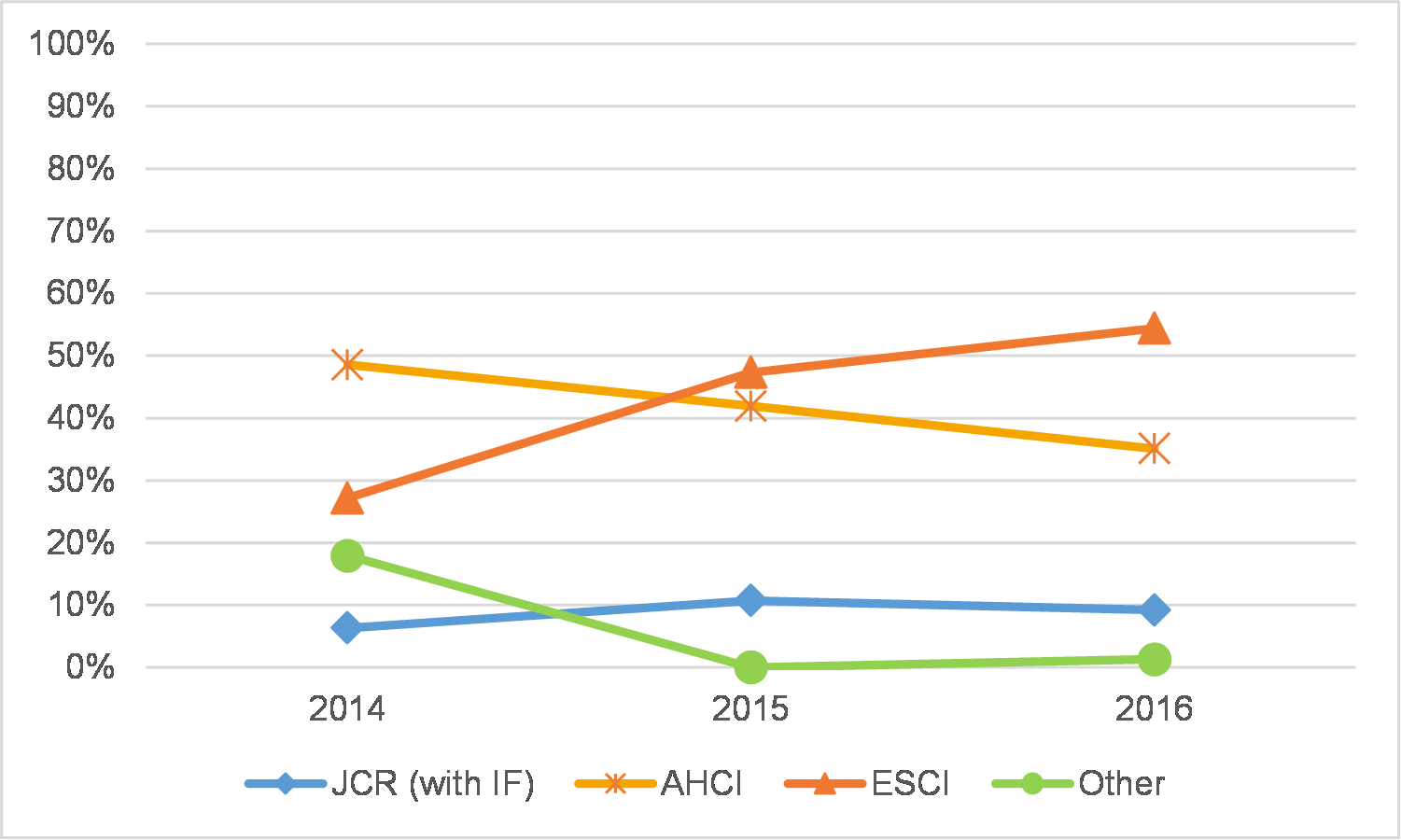

Supplement: S7 Fig — Slovakia–humanities. (TIF) [file pone.0249879.s007.tif]

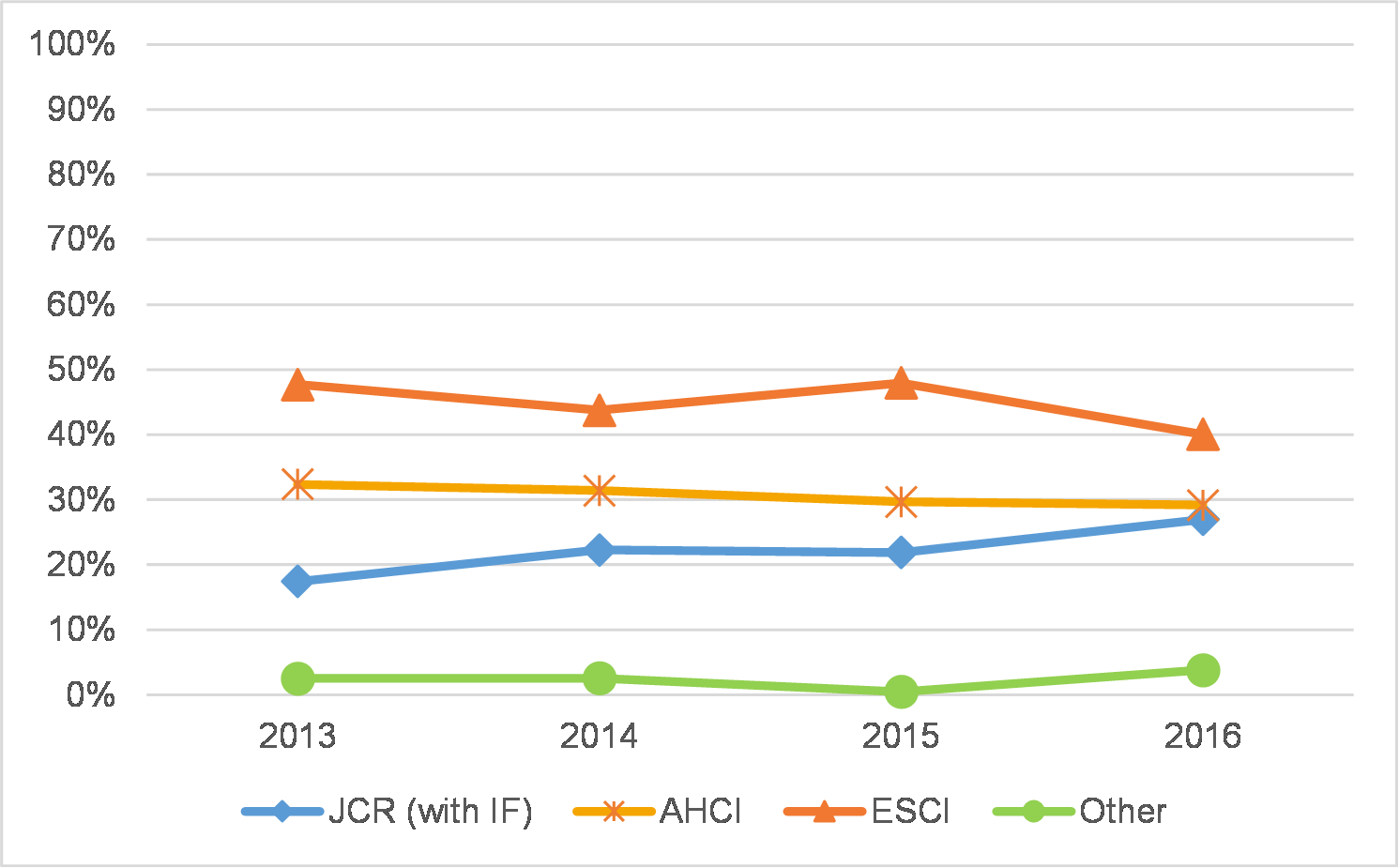

Supplement: S8 Fig — Poland–humanities. (TIF) [file pone.0249879.s008.tif]

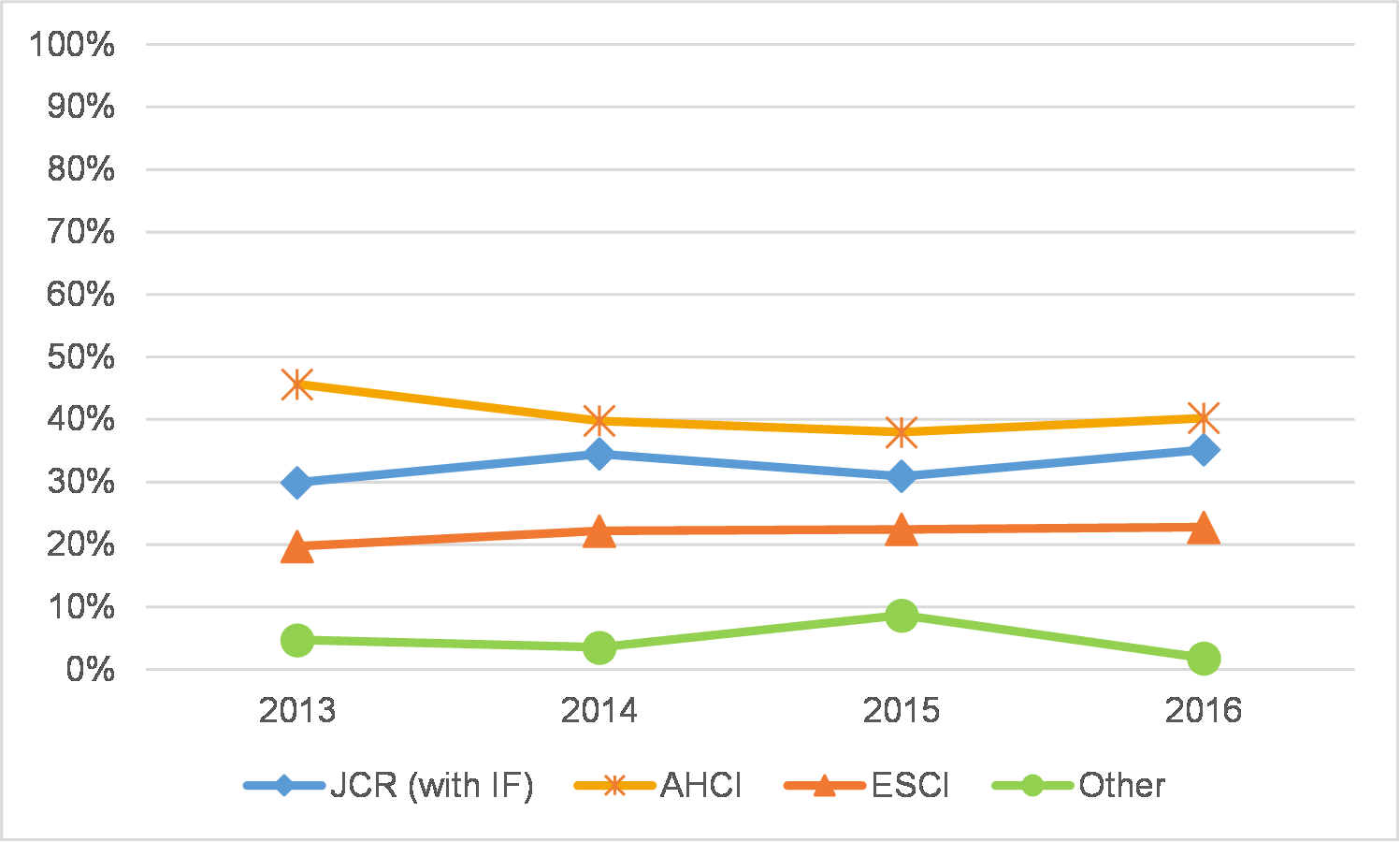

Supplement: S9 Fig — Norway–humanities. (TIF) [file pone.0249879.s009.tif]

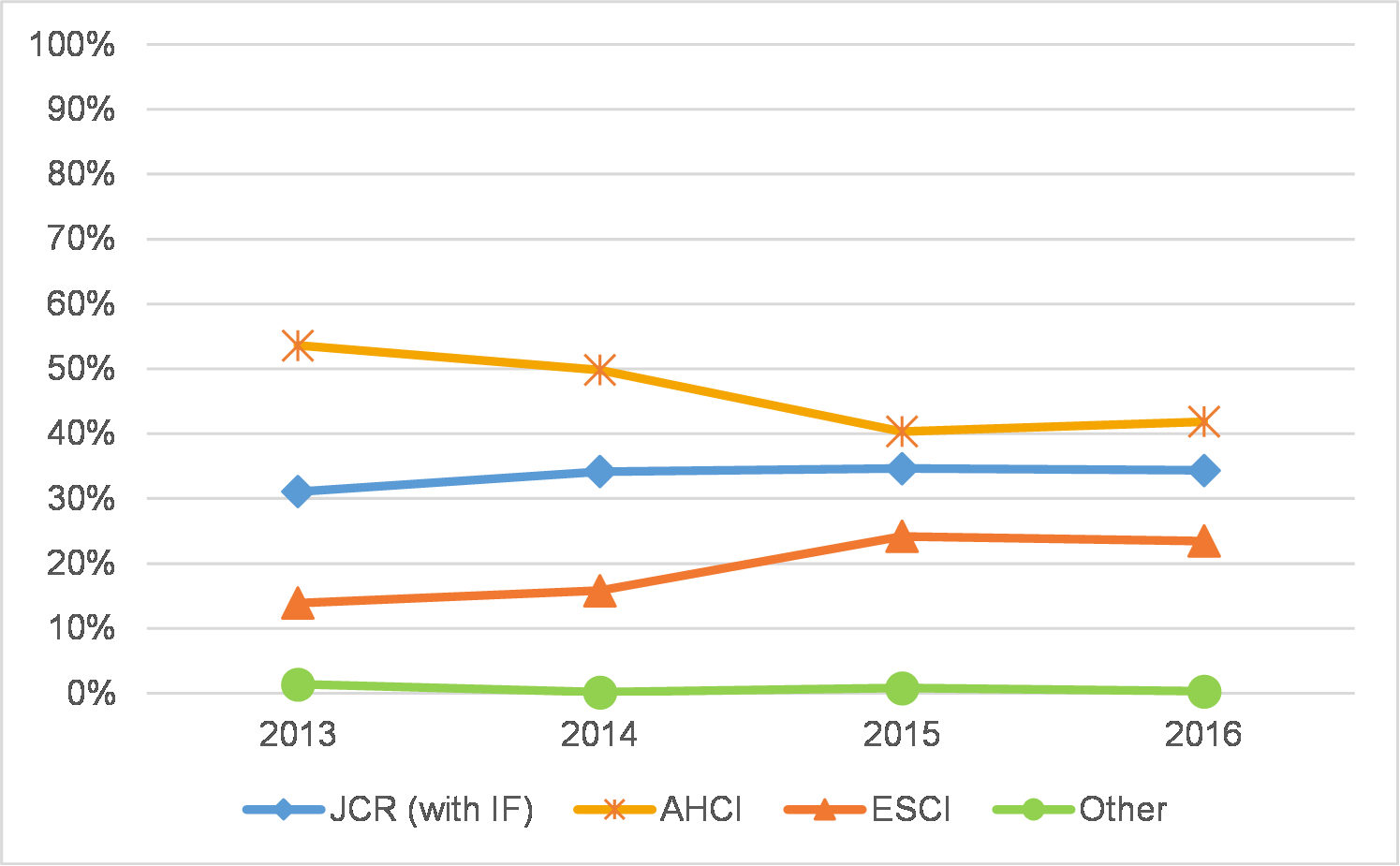

Supplement: S10 Fig — Flanders–humanities. (TIF) [file pone.0249879.s010.tif]
