# Supplementary material for: Journal article publishing in the social sciences and humanities: A comparison of Web of Science coverage for five European countries
Source: PLoS One. 2021 Apr 8;16(4):e0249879. doi: 10.1371/journal.pone.0249879 (PMC8031415; doi:10.1371/journal.pone.0249879)
Supplement: S1 Table — (DOCX) [file pone.0249879.s011.docx]

**S1 Table. Article share – social sciences.**

|  | 2013 | | 2014 | | 2015 | | 2016 | |
| --- | --- | --- | --- | --- | --- | --- | --- | --- |
|  | # | % | # | % | # | % | # | % |
|  | Psychology | | | | | | | |
| CZE | 237 | 46.5% | 217 | 41.8% | 249 | 50.9% | 319 | 54.9% |
| SLO | n/a |  | n/a |  | n/a |  | n/a |  |
| POL | 1,003 | 60.3% | 1,114 | 57.7% | 1,150 | 57.8% | 1,159 | 59.3% |
| NOR | 534 | 86.7% | 549 | 84.6% | 544 | 86.3% | 617 | 84.5% |
| FLA | 555 | 90.5% | 586 | 94.1% | 688 | 91.7% | 623 | 90.4% |
|  | Economics and business | | | | | | | |
| CZE | 659 | 37.5% | 584 | 34.2% | 579 | 34.0% | 622 | 38.8% |
| SLO | n/a |  | 1,501 | 30.5% | 1,441 | 31.0% | 1,360 | 30.9% |
| POL | 10,260 | 56.0% | 10,780 | 56.0% | 10,167 | 56.7% | 9,651 | 54.8% |
| NOR | 718 | 73.6% | 744 | 73.4% | 791 | 78.9% | 897 | 80.6% |
| FLA | 464 | 71.2% | 488 | 68.7% | 520 | 70.9% | 526 | 68.4% |
|  | Educational sciences | | | | | | | |
| CZE | 815 | 35.4% | 771 | 35.6% | 789 | 39.1% | 744 | 41.9% |
| SLO | n/a |  | 547 | 21.1% | 607 | 26.7% | 602 | 28.1% |
| POL | 1,426 | 33.3% | 1,685 | 33.7% | 1,706 | 32.6% | 1,568 | 32.9% |
| NOR | 424 | 46.8% | 492 | 45.9% | 527 | 53.4% | 588 | 54.8% |
| FLA | 167 | 71.4% | 192 | 69.6% | 227 | 67.6% | 207 | 60.9% |
|  | Sociology | | | | | | | |
| CZE | 410 | 44.8% | 409 | 47.3% | 443 | 53.8% | 382 | 51.6% |
| SLO | n/a |  | n/a |  | n/a |  | n/a |  |
| POL | 1,144 | 38.3% | 1,282 | 38.9% | 1,281 | 36.6% | 1,057 | 35.0% |
| NOR | 335 | 71.7% | 365 | 66.2% | 388 | 72.3% | 467 | 77.8% |
| FLA | 264 | 58.0% | 282 | 62.5% | 319 | 55.0% | 295 | 51.7% |
|  | Law |  |  |  |  |  |  |  |
| CZE | 681 | 46.1% | 899 | 49.2% | 737 | 44.3% | 773 | 46.8% |
| SLO | n/a |  | 309 | 18.5% | 375 | 19.7% | 450 | 21.6% |
| POL | 3,406 | 40.6% | 3,691 | 39.9% | 3,507 | 37.6% | 2,925 | 30.8% |
| NOR | 209 | 44.2% | 219 | 53.4% | 216 | 51.6% | 207 | 40.0% |
| FLA | 739 | 72.7% | 800 | 69.3% | 929 | 63.2% | 854 | 63.4% |
|  | Political science | | | | | | | |
| CZE | 1,273 | 45.8% | 1,118 | 37.5% | 1,238 | 40.8% | 1,220 | 47.0% |
| SLO | n/a |  | n/a |  | n/a |  | n/a |  |
| POL | 1,250 | 36.0% | 1,336 | 34.4% | 1,429 | 37.6% | 1,222 | 33.0% |
| NOR | 284 | 54.4% | 269 | 51.3% | 310 | 53.1% | 287 | 50.5% |
| FLA | 179 | 64.4% | 203 | 66.1% | 213 | 55.6% | 231 | 54.9% |
|  | Social and economic geography | | | | | | | |
| CZE | 45 | 34.1% | 41 | 36.3% | 44 | 32.6% | 48 | 34.8% |
| SLO | n/a |  | n/a |  | n/a |  | n/a |  |
| POL | n/a |  | n/a |  | n/a |  | n/a |  |
| NOR | 270 | 64.3% | 286 | 68.3% | 266 | 59.4% | 306 | 65.5% |
| FLA | 191 | 85.3% | 213 | 89.9% | 231 | 88.2% | 222 | 73.3% |
|  | Media and communication | | | | | | | |
| CZE | 104 | 49.8% | 116 | 45.0% | 87 | 39.2% | 91 | 39.9% |
| SLO | n/a |  | n/a |  | n/a |  | n/a |  |
| POL | 329 | 38.9% | 331 | 38.7% | 319 | 33.6% | 267 | 28.0% |
| NOR | 103 | 55.4% | 95 | 43.4% | 100 | 45.0% | 121 | 48.8% |
| FLA | 126 | 76.4% | 134 | 71.3% | 160 | 78.0% | 150 | 70.8% |
|  | Other social sciences | | | | | | | |
| CZE | 149 | 44.5% | 156 | 44.7% | 163 | 68.2% | 199 | 58.7% |
| SLO | n/a |  | n/a |  | n/a |  | n/a |  |
| POL | 719 | 42.6% | 792 | 39.7% | 777 | 34.4% | 679 | 33.6% |
| NOR | 404 | 59.1% | 403 | 65.4% | 503 | 62.4% | 503 | 71.7% |
| FLA | 44 | 62.0% | 45 | 47.4% | 53 | 47.7% | 72 | 53.7% |

CZE Czech Republic, SLO Slovakia, POL Poland, NOR Norway, FLA Flanders
