# Supplementary material for: Journal article publishing in the social sciences and humanities: A comparison of Web of Science coverage for five European countries
Source: PLoS One. 2021 Apr 8;16(4):e0249879. doi: 10.1371/journal.pone.0249879 (PMC8031415; doi:10.1371/journal.pone.0249879)
Supplement: S2 Table — (DOCX) [file pone.0249879.s012.docx]

**S2 Table. Article share – humanities.**

|  | 2013 | | 2014 | | 2015 | | 2016 | |
| --- | --- | --- | --- | --- | --- | --- | --- | --- |
|  | # | % | # | % | # | % | # | % |
|  | History and archaeology | | |  |  |  |  |  |
| CZE | 1,155 | 45.7% | 1,298 | 44.9% | 1,252 | 45.7% | 1,162 | 44.3% |
| SLO | n/a |  | 182 | 23.8% | 196 | 28.7% | 226 | 32.9% |
| POL | 2,359 | 39.2% | 2,443 | 35.1% | 2,393 | 34.7% | 1,784 | 28.3% |
| NOR | 223 | 46.1% | 215 | 36.3% | 287 | 51.3% | 236 | 48.9% |
| FLA | 273 | 79.1% | 270 | 67.2% | 313 | 72.1% | 334 | 71.7% |
|  | Languages and literature | | |  |  |  |  |  |
| CZE | 808 | 42.3% | 815 | 40.4% | 810 | 40.0% | 828 | 47.6% |
| SLO | n/a |  | n/a |  | n/a |  | n/a |  |
| POL | 4,270 | 42.2% | 4,569 | 37.6% | 4,667 | 35.9% | 3,801 | 31.6% |
| NOR | 422 | 55.0% | 508 | 56.3% | 449 | 61.0% | 435 | 56.5% |
| FLA | 437 | 49.0% | 429 | 57.7% | 494 | 59.5% | 474 | 53.8% |
|  | Philosophy, ethics and religion | | | | | | | |
| CZE | 475 | 49.2% | 588 | 55.1% | 507 | 50.0% | 482 | 51.7% |
| SLO | n/a |  | n/a |  | n/a |  | n/a |  |
| POL | 1,995 | 46.8% | 1,968 | 45.2% | 2,060 | 44.5% | 1,771 | 42.8% |
| NOR | 281 | 48.0% | 324 | 58.5% | 353 | 57.1% | 292 | 53.0% |
| FLA | 241 | 50.8% | 291 | 52.9% | 307 | 53.9% | 303 | 48.5% |
|  | Arts |  |  |  |  |  |  |  |
| CZE | 526 | 39.0% | 617 | 40.8% | 615 | 40.1% | 558 | 44.1% |
| SLO | n/a |  | 27 | 20.1% | 49 | 27.1% | 39 | 23.8% |
| POL | 541 | 44.6% | 585 | 41.0% | 492 | 35.5% | 363 | 25.5% |
| NOR | 154 | 59.9% | 172 | 59.9% | 185 | 66.3% | 153 | 57.3% |
| FLA | 107 | 43.7% | 120 | 47.8% | 147 | 45.1% | 147 | 51.4% |
|  | Other humanities | | | | | | | |
| CZE | 0 |  | 0 |  | 0 |  | 6 | 40.0% |
| SLO | n/a |  | 998 | 25.1% | 1,104 | 29.0% | 1,038 | 29.4% |
| POL | 510 | 46.2% | 530 | 38.0% | 546 | 36.9% | 406 | 36.2% |
| NOR | 102 | 65.4% | 97 | 48.3% | 150 | 63.6% | 89 | 63.1% |
| FLA | 71 | 93.4% | 94 | 96.9% | 105 | 98.1% | 106 | 96.4% |

CZE Czech Republic, SLO Slovakia, POL Poland, NOR Norway, FLA Flanders
