# Supplementary material for: Journal article publishing in the social sciences and humanities: A comparison of Web of Science coverage for five European countries
Source: PLoS One. 2021 Apr 8;16(4):e0249879. doi: 10.1371/journal.pone.0249879 (PMC8031415; doi:10.1371/journal.pone.0249879)
Supplement: S3 Table — (DOCX) [file pone.0249879.s013.docx]

**S3 Table. Coverage of journal articles in WoS – social sciences.**

|  | 2013 | | 2014 | | 2015 | | 2016 | |
| --- | --- | --- | --- | --- | --- | --- | --- | --- |
|  | # | % | # | % | # | % | # | % |
|  | Psychology | |  |  |  |  |  |  |
| CZE | 85 | 35.9% | 94 | 43.3% | 117 | 47.0% | 149 | 46.7% |
| SLO | n/a |  | n/a |  | n/a |  | n/a |  |
| POL | 351 | 35.0% | 424 | 38.1% | 469 | 40.8% | 535 | 46.2% |
| NOR | 411 | 77.3% | 434 | 79.5% | 426 | 78.3% | 482 | 78.1% |
| FLA | 503 | 91.0% | 532 | 92.8% | 506 | 90.8% | 560 | 90.5% |
|  | Economics and business | | |  |  |  |  |  |
| CZE | 192 | 29.1% | 220 | 37.7% | 249 | 43.0% | 292 | 47.0% |
| SLO | n/a |  | 149 | 10.2% | 185 | 13.3% | 242 | 18.3% |
| POL | 669 | 6.5% | 828 | 7.7% | 1,073 | 10.6% | 981 | 10.2% |
| NOR | 511 | 71.5% | 539 | 72.4% | 597 | 75.5% | 657 | 73.2% |
| FLA | 380 | 81.7% | 375 | 80.3% | 346 | 86.9% | 420 | 80.2% |
|  | Educational sciences | | | | | | | |
| CZE | 74 | 9.1% | 72 | 9.3% | 95 | 12.0% | 93 | 12.5% |
| SLO | n/a |  | 65 | 11.9% | 49 | 8.2% | 100 | 16.9% |
| POL | 47 | 3.3% | 68 | 4.0% | 75 | 4.4% | 97 | 6.2% |
| NOR | 166 | 39.1% | 216 | 43.9% | 231 | 44.0% | 273 | 46.4% |
| FLA | 125 | 74.9% | 126 | 67.0% | 138 | 73.8% | 145 | 71.4% |
|  | Sociology | | | | | | | |
| CZE | 122 | 29.8% | 111 | 27.2% | 134 | 30.3% | 131 | 34.2% |
| SLO | n/a |  | n/a |  | n/a |  | n/a |  |
| POL | 115 | 10.1% | 115 | 9.0% | 129 | 10.1% | 113 | 10.7% |
| NOR | 222 | 66.9% | 240 | 65.8% | 273 | 70.4% | 295 | 63.2% |
| FLA | 171 | 65.8% | 182 | 68.7% | 204 | 77.3% | 186 | 63.1% |
|  | Law |  |  |  |  |  |  |  |
| CZE | 18 | 2.6% | 24 | 2.7% | 21 | 2.8% | 29 | 3.8% |
| SLO | n/a |  | 3 | 1.0% | 15 | 4.1% | 14 | 3.2% |
| POL | 57 | 1.7% | 87 | 2.4% | 130 | 3.7% | 109 | 3.7% |
| NOR | 54 | 25.8% | 63 | 28.9% | 55 | 25.5% | 53 | 25.6% |
| FLA | 90 | 12.6% | 122 | 15.9% | 112 | 16.3% | 124 | 15.2% |
|  | Political science | |  |  |  |  |  |  |
| CZE | 222 | 17.4% | 220 | 19.7% | 328 | 26.5% | 332 | 27.2% |
| SLO | n/a |  | n/a |  | n/a |  | n/a |  |
| POL | 34 | 2.7% | 46 | 3.4% | 68 | 4.8% | 57 | 4.7% |
| NOR | 210 | 75.5% | 211 | 77.6% | 255 | 82.5% | 218 | 76.0% |
| FLA | 94 | 53.4% | 119 | 60.1% | 102 | 65.8% | 128 | 55.7% |
|  | Social and economic geography | | | | | | | |
| CZE | 5 | 11.1% | 10 | 24.4% | 16 | 36.4% | 17 | 35.4% |
| SLO | n/a |  | n/a |  | n/a |  | n/a |  |
| POL | n/a |  | n/a |  | n/a |  | n/a |  |
| NOR | 237 | 87.8% | 246 | 86.3% | 227 | 85.7% | 268 | 87.6% |
| FLA | 164 | 87.2% | 178 | 85.2% | 148 | 86.0% | 195 | 87.8% |
|  | Media and communication studies | | |  |  |  |  |  |
| CZE | 20 | 19.2% | 25 | 21.6% | 26 | 29.9% | 16 | 17.6% |
| SLO | n/a |  | n/a |  | n/a |  | n/a |  |
| POL | 9 | 2.7% | 14 | 4.2% | 17 | 5.3% | 10 | 3.7% |
| NOR | 62 | 60.2% | 58 | 61.1% | 62 | 62.0% | 72 | 59.5% |
| FLA | 77 | 62.1% | 91 | 68.9% | 104 | 71.2% | 92 | 61.7% |
|  | Other social sciences | | |  |  |  |  |  |
| CZE | 27 | 18.1% | 64 | 41.0% | 82 | 50.3% | 84 | 42.4% |
| SLO | n/a |  | 75 | 16.0% | 74 | 14.8% | 150 | 28.2% |
| POL | 71 | 9.9% | 46 | 5.8% | 95 | 12.2% | 51 | 7.5% |
| NOR | 211 | 52.2% | 206 | 51.2% | 259 | 51.5% | 274 | 54.5% |
| FLA | 21 | 47.7% | 23 | 51.1% | 22 | 64.7% | 35 | 47.3% |

CZE Czech Republic, SLO Slovakia, POL Poland, NOR Norway, FLA Flanders
