# Supplementary material for: Journal article publishing in the social sciences and humanities: A comparison of Web of Science coverage for five European countries
Source: PLoS One. 2021 Apr 8;16(4):e0249879. doi: 10.1371/journal.pone.0249879 (PMC8031415; doi:10.1371/journal.pone.0249879)
Supplement: S4 Table — (DOCX) [file pone.0249879.s014.docx]

**S4 Table. Coverage of journal articles in WoS – humanities.**

|  | 2013 | | 2014 | | 2015 | | 2016 | |
| --- | --- | --- | --- | --- | --- | --- | --- | --- |
|  | # | % | # | % | # | % | # | % |
|  | History and archaeology | | |  |  |  |  |  |
| CZE | 134 | 11.6% | 133 | 10.2% | 135 | 10.8% | 154 | 13.3% |
| SLO | n/a |  | 26 | 14.4% | 28 | 14.4% | 31 | 13.7% |
| POL | 209 | 8.9% | 156 | 6.4% | 147 | 6.1% | 135 | 7.6% |
| NOR | 92 | 41.3% | 89 | 41.4% | 111 | 38.7% | 102 | 43.2% |
| FLA | 141 | 52.2% | 136 | 50.6% | 95 | 44.2% | 164 | 49.1% |
|  | Languages and literature | | | | | | | |
| CZE | 99 | 12.2% | 106 | 13.1% | 114 | 14.1% | 132 | 15.9% |
| SLO | n/a |  | n/a |  | n/a |  | n/a |  |
| POL | 342 | 8.0% | 360 | 7.9% | 460 | 9.9% | 355 | 9.3% |
| NOR | 138 | 32.7% | 136 | 26.8% | 149 | 33.3% | 171 | 39.3% |
| FLA | 263 | 60.6% | 246 | 57.6% | 213 | 60.0% | 253 | 53.5% |
|  | Philosophy, ethics and religion | | | |  |  |  |  |
| CZE | 153 | 32.2% | 197 | 33.8% | 169 | 33.2% | 165 | 34.2% |
| SLO | n/a |  | n/a |  | n/a |  | n/a |  |
| POL | 140 | 7.0% | 143 | 7.3% | 162 | 7.9% | 184 | 10.4% |
| NOR | 102 | 36.4% | 107 | 33.2% | 137 | 38.8% | 112 | 38.4% |
| FLA | 133 | 56.6% | 157 | 54.9% | 117 | 53.9% | 136 | 45.5% |
|  | Arts | | | | | | | |
| CZE | 79 | 15.0% | 92 | 15.0% | 112 | 18.2% | 101 | 18.1% |
| SLO | n/a |  | 1 | 3.7% | 1 | 2.0% | 5 | 12.8% |
| POL | 33 | 6.1% | 53 | 9.1% | 34 | 6.9% | 28 | 7.7% |
| NOR | 52 | 33.8% | 61 | 35.7% | 82 | 44.6% | 79 | 51.6% |
| FLA | 61 | 59.2% | 54 | 46.6% | 56 | 54.4% | 60 | 40.8% |
|  | Other humanities | | | |  |  |  |  |
| CZE | 0 |  | 0 |  | 0 |  | 1 | 16.7% |
| SLO | n/a |  | 146 | 14.7% | 157 | 14.5% | 192 | 18.6% |
| POL | 33 | 6.5% | 33 | 6.2% | 33 | 6.0% | 32 | 7.9% |
| NOR | 18 | 17.6% | 9 | 9.3% | 20 | 13.3% | 28 | 31.5% |
| FLA | 15 | 51.7% | 26 | 66.7% | 21 | 61.8% | 34 | 57.6% |

CZE Czech Republic, SLO Slovakia, POL Poland, NOR Norway, FLA Flanders
