# Supplementary material for: Journal article publishing in the social sciences and humanities: A comparison of Web of Science coverage for five European countries
Source: PLoS One. 2021 Apr 8;16(4):e0249879. doi: 10.1371/journal.pone.0249879 (PMC8031415; doi:10.1371/journal.pone.0249879)
Supplement: S5 Table — (DOCX) [file pone.0249879.s015.docx]

**S5 Table. Proportion of articles in WoS by citation indexes.**

|  | year | Q1+Q2 | | Q3+Q4 | | AHCI | | ESCI | | Other | |
| --- | --- | --- | --- | --- | --- | --- | --- | --- | --- | --- | --- |
|  |  | # | % | # | % | # | % | # | % | # | % |
|  | Psychology | | | | | | | | | | |
| CZE |  |  |  |  |  |  |  |  |  |  |  |
|  | 2013 | 29 | 34.1% | 44 | 51.8% | 0 | 0.0% | 8 | 9.4% | 4 | 4.7% |
|  | 2014 | 29 | 30.9% | 55 | 58.5% | 0 | 0.0% | 5 | 5.3% | 5 | 5.3% |
|  | 2015 | 44 | 37.6% | 60 | 51.3% | 0 | 0.0% | 10 | 8.5% | 3 | 2.6% |
|  | 2016 | 63 | 42.3% | 68 | 45.6% | 0 | 0.0% | 14 | 9.4% | 4 | 2.7% |
| SLO |  | n/a |  | n/a |  | n/a |  | n/a |  | n/a |  |
| POL |  |  |  |  |  |  |  |  |  |  |  |
|  | 2013 | 126 | 35.9% | 149 | 42.5% | 3 | 0.9% | 64 | 18.2% | 9 | 2.6% |
|  | 2014 | 157 | 37.0% | 135 | 31.8% | 3 | 0.7% | 126 | 29.7% | 3 | 0.7% |
|  | 2015 | 219 | 46.7% | 146 | 31.1% | 2 | 0.4% | 97 | 20.7% | 5 | 1.1% |
|  | 2016 | 270 | 50.5% | 151 | 28.2% | 3 | 0.6% | 107 | 20.0% | 4 | 0.7% |
| NOR |  |  |  |  |  |  |  |  |  |  |  |
|  | 2013 | 252 | 61.3% | 107 | 26.0% | 0 | 0.0% | 45 | 10.9% | 7 | 1.7% |
|  | 2014 | 283 | 65.2% | 114 | 26.3% | 0 | 0.0% | 32 | 7.4% | 5 | 1.2% |
|  | 2015 | 287 | 67.4% | 105 | 24.6% | 0 | 0.0% | 33 | 7.7% | 1 | 0.2% |
|  | 2016 | 332 | 68.9% | 105 | 21.8% | 0 | 0.0% | 44 | 9.1% | 1 | 0.2% |
| FLA |  |  |  |  |  |  |  |  |  |  |  |
|  | 2013 | 397 | 78.9% | 85 | 16.9% | 0 | 0.0% | 11 | 2.2% | 10 | 2.0% |
|  | 2014 | 433 | 81.4% | 87 | 16.4% | 1 | 0.2% | 10 | 1.9% | 1 | 0.2% |
|  | 2015 | 427 | 84.4% | 60 | 11.9% | 0 | 0.0% | 17 | 3.4% | 2 | 0.4% |
|  | 2016 | 430 | 76.8% | 112 | 20.0% | 0 | 0.0% | 17 | 3.0% | 1 | 0.2% |
|  | Economics and business | | | | | | | | | | |
| CZE |  |  |  |  |  |  |  |  |  |  |  |
|  | 2013 | 44 | 22.9% | 125 | 65.1% | 0 | 0.0% | 20 | 10.4% | 3 | 1.6% |
|  | 2014 | 68 | 30.9% | 108 | 49.1% | 0 | 0.0% | 34 | 15.5% | 10 | 4.5% |
|  | 2015 | 96 | 38.6% | 94 | 37.8% | 0 | 0.0% | 54 | 21.7% | 5 | 2.0% |
|  | 2016 | 78 | 26.7% | 122 | 41.8% | 0 | 0.0% | 87 | 29.8% | 5 | 1.7% |
| SLO |  |  |  |  |  |  |  |  |  |  |  |
|  | 2014 | 21 | 14.1% | 68 | 45.6% | 0 | 0.0% | 50 | 33.6% | 10 | 6.7% |
|  | 2015 | 38 | 20.5% | 80 | 43.2% | 0 | 0.0% | 65 | 35.1% | 2 | 1.1% |
|  | 2016 | 44 | 18.2% | 66 | 27.3% | 0 | 0.0% | 130 | 53.7% | 2 | 0.8% |
| POL |  |  |  |  |  |  |  |  |  |  |  |
|  | 2013 | 135 | 20.2% | 202 | 30.2% | 1 | 0.1% | 293 | 43.8% | 38 | 5.7% |
|  | 2014 | 175 | 21.1% | 177 | 21.4% | 2 | 0.2% | 378 | 45.7% | 96 | 11.6% |
|  | 2015 | 187 | 17.4% | 281 | 26.2% | 3 | 0.3% | 479 | 44.6% | 123 | 11.5% |
|  | 2016 | 226 | 23.0% | 286 | 29.2% | 3 | 0.3% | 396 | 40.4% | 70 | 7.1% |
| NOR |  |  |  |  |  |  |  |  |  |  |  |
|  | 2013 | 270 | 52.8% | 114 | 22.3% | 0 | 0.0% | 106 | 20.7% | 21 | 4.1% |
|  | 2014 | 280 | 51.9% | 128 | 23.7% | 1 | 0.2% | 112 | 20.8% | 18 | 3.3% |
|  | 2015 | 303 | 50.8% | 130 | 21.8% | 0 | 0.0% | 123 | 20.6% | 41 | 6.9% |
|  | 2016 | 361 | 54.9% | 115 | 17.5% | 0 | 0.0% | 150 | 22.8% | 31 | 4.7% |
| FLA |  |  |  |  |  |  |  |  |  |  |  |
|  | 2013 | 226 | 59.5% | 95 | 25.0% | 0 | 0.0% | 42 | 11.1% | 17 | 4.5% |
|  | 2014 | 205 | 54.7% | 86 | 22.9% | 1 | 0.3% | 75 | 20.0% | 8 | 2.1% |
|  | 2015 | 204 | 59.0% | 83 | 24.0% | 1 | 0.3% | 56 | 16.2% | 2 | 0.6% |
|  | 2016 | 266 | 63.3% | 108 | 25.7% | 0 | 0.0% | 43 | 10.2% | 3 | 0.7% |
|  | Educational sciences | | | | | | | | | | |
| CZE |  |  |  |  |  |  |  |  |  |  |  |
|  | 2013 | 10 | 13.5% | 16 | 21.6% | 2 | 2.7% | 34 | 45.9% | 12 | 16.2% |
|  | 2014 | 11 | 15.3% | 17 | 23.6% | 2 | 2.8% | 34 | 47.2% | 8 | 11.1% |
|  | 2015 | 15 | 15.8% | 21 | 22.1% | 2 | 2.1% | 38 | 40.0% | 19 | 20.0% |
|  | 2016 | 17 | 18.3% | 13 | 14.0% |  | 0.0% | 52 | 55.9% | 11 | 11.8% |
| SLO |  |  |  |  |  |  |  |  |  |  |  |
|  | 2014 | 18 | 27.7% | 20 | 30.8% | 5 | 7.7% | 18 | 27.7% | 4 | 6.2% |
|  | 2015 | 11 | 22.4% | 11 | 22.4% | 2 | 4.1% | 25 | 51.0% | 0 | 0.0% |
|  | 2016 | 28 | 28.0% | 24 | 24.0% | 2 | 2.0% | 45 | 45.0% | 1 | 1.0% |
| POL |  |  |  |  |  |  |  |  |  |  |  |
|  | 2013 | 12 | 25.5% | 12 | 25.5% | 0 | 0.0% | 18 | 38.3% | 5 | 10.6% |
|  | 2014 | 7 | 10.3% | 20 | 29.4% | 1 | 1.5% | 38 | 55.9% | 2 | 2.9% |
|  | 2015 | 13 | 17.3% | 17 | 22.7% | 0 | 0.0% | 43 | 57.3% | 2 | 2.7% |
|  | 2016 | 25 | 25.8% | 23 | 23.7% | 1 | 1.0% | 45 | 46.4% | 3 | 3.1% |
| NOR |  |  |  |  |  |  |  |  |  |  |  |
|  | 2013 | 41 | 24.7% | 54 | 32.5% | 0 | 0.0% | 60 | 36.1% | 11 | 6.6% |
|  | 2014 | 47 | 21.8% | 58 | 26.9% | 1 | 0.5% | 88 | 40.7% | 22 | 10.2% |
|  | 2015 | 67 | 29.0% | 60 | 26.0% | 0 | 0.0% | 77 | 33.3% | 27 | 11.7% |
|  | 2016 | 58 | 21.2% | 82 | 30.0% | 0 | 0.0% | 106 | 38.8% | 27 | 9.9% |
| FLA |  |  |  |  |  |  |  |  |  |  |  |
|  | 2013 | 46 | 36.8% | 47 | 37.6% | 1 | 0.8% | 30 | 24.0% | 1 | 0.8% |
|  | 2014 | 60 | 47.6% | 37 | 29.4% | 1 | 0.8% | 26 | 20.6% | 2 | 1.6% |
|  | 2015 | 62 | 44.9% | 45 | 32.6% | 1 | 0.7% | 30 | 21.7% | 0 | 0.0% |
|  | 2016 | 63 | 43.4% | 44 | 30.3% | 1 | 0.7% | 37 | 25.5% | 0 | 0.0% |
|  | Sociology | | | | | | | | | | |
| CZE |  |  |  |  |  |  |  |  |  |  |  |
|  | 2013 | 25 | 20.5% | 47 | 38.5% | 2 | 1.6% | 31 | 25.4% | 17 | 13.9% |
|  | 2014 | 27 | 24.3% | 54 | 48.6% | 2 | 1.8% | 27 | 24.3% | 1 | 0.9% |
|  | 2015 | 31 | 23.1% | 56 | 41.8% | 3 | 2.2% | 42 | 31.3% | 2 | 1.5% |
|  | 2016 | 31 | 23.7% | 43 | 32.8% | 6 | 4.6% | 51 | 38.9% | 0 | 0.0% |
| SLO |  | n/a |  | n/a |  | n/a |  | n/a |  | n/a |  |
| POL |  |  |  |  |  |  |  |  |  |  |  |
|  | 2013 | 21 | 18.3% | 53 | 46.1% | 5 | 4.3% | 31 | 27.0% | 5 | 4.3% |
|  | 2014 | 37 | 32.2% | 46 | 40.0% | 5 | 4.3% | 22 | 19.1% | 5 | 4.3% |
|  | 2015 | 32 | 24.8% | 52 | 40.3% | 6 | 4.7% | 38 | 29.5% | 1 | 0.8% |
|  | 2016 | 27 | 23.9% | 40 | 35.4% | 3 | 2.7% | 42 | 37.2% | 1 | 0.9% |
| NOR |  |  |  |  |  |  |  |  |  |  |  |
|  | 2013 | 97 | 43.7% | 44 | 19.8% | 0 | 0.0% | 55 | 24.8% | 26 | 11.7% |
|  | 2014 | 101 | 42.1% | 53 | 22.1% | 0 | 0.0% | 67 | 27.9% | 19 | 7.9% |
|  | 2015 | 127 | 46.5% | 40 | 14.7% | 0 | 0.0% | 73 | 26.7% | 33 | 12.1% |
|  | 2016 | 129 | 43.7% | 55 | 18.6% | 0 | 0.0% | 85 | 28.8% | 26 | 8.8% |
| FLA |  |  |  |  |  |  |  |  |  |  |  |
|  | 2013 | 99 | 57.9% | 45 | 26.3% | 7 | 4.1% | 19 | 11.1% | 1 | 0.6% |
|  | 2014 | 102 | 56.0% | 43 | 23.6% | 5 | 2.7% | 32 | 17.6% | 0 | 0.0% |
|  | 2015 | 112 | 54.9% | 47 | 23.0% | 1 | 0.5% | 44 | 21.6% | 0 | 0.0% |
|  | 2016 | 90 | 48.4% | 63 | 33.9% | 5 | 2.7% | 28 | 15.1% | 0 | 0.0% |
|  | Law |  |  |  |  |  |  |  |  |  |  |
| CZE |  |  |  |  |  |  |  |  |  |  |  |
|  | 2013 | 1 | 5.6% | 1 | 5.6% | 0 | 0.0% | 16 | 88.9% | 0 | 0.0% |
|  | 2014 | 2 | 8.3% | 6 | 25.0% | 1 | 4.2% | 11 | 45.8% | 4 | 16.7% |
|  | 2015 | 2 | 9.5% | 3 | 14.3% | 0 | 0.0% | 15 | 71.4% | 1 | 4.8% |
|  | 2016 | 4 | 13.8% | 5 | 17.2% | 3 | 10.3% | 16 | 55.2% | 1 | 3.4% |
| SLO |  |  |  |  |  |  |  |  |  |  |  |
|  | 2014 | 0 | 0.0% | 0 | 0.0% | 1 | 33.3% | 1 | 33.3% | 1 | 33.3% |
|  | 2015 | 2 | 13.3% | 2 | 13.3% | 2 | 13.3% | 9 | 60.0% | 0 | 0.0% |
|  | 2016 | 0 | 0.0% | 2 | 14.3% | 2 | 14.3% | 10 | 71.4% | 0 | 0.0% |
| POL |  |  |  |  |  |  |  |  |  |  |  |
|  | 2013 | 11 | 19.3% | 17 | 29.8% | 3 | 5.3% | 24 | 42.1% | 2 | 3.5% |
|  | 2014 | 10 | 11.5% | 29 | 33.3% | 0 | 0.0% | 47 | 54.0% | 1 | 1.1% |
|  | 2015 | 14 | 10.8% | 26 | 20.0% | 1 | 0.8% | 88 | 67.7% | 1 | 0.8% |
|  | 2016 | 14 | 12.8% | 18 | 16.5% | 9 | 8.3% | 66 | 60.6% | 2 | 1.8% |
| NOR |  |  |  |  |  |  |  |  |  |  |  |
|  | 2013 | 15 | 27.8% | 12 | 22.2% | 0 | 0.0% | 23 | 42.6% | 4 | 7.4% |
|  | 2014 | 11 | 17.5% | 27 | 42.9% | 0 | 0.0% | 24 | 38.1% | 1 | 1.6% |
|  | 2015 | 17 | 30.9% | 14 | 25.5% | 0 | 0.0% | 23 | 41.8% | 1 | 1.8% |
|  | 2016 | 12 | 22.6% | 11 | 20.8% | 0 | 0.0% | 30 | 56.6% | 0 | 0.0% |
| FLA |  |  |  |  |  |  |  |  |  |  |  |
|  | 2013 | 12 | 13.3% | 19 | 21.1% | 0 | 0.0% | 55 | 61.1% | 4 | 4.4% |
|  | 2014 | 23 | 18.9% | 27 | 22.1% | 0 | 0.0% | 72 | 59.0% | 0 | 0.0% |
|  | 2015 | 23 | 20.5% | 17 | 15.2% | 0 | 0.0% | 72 | 64.3% | 0 | 0.0% |
|  | 2016 | 35 | 28.2% | 22 | 17.7% | 0 | 0.0% | 67 | 54.0% | 0 | 0.0% |
|  | Political science | | | | | | | | | | |
| CZE |  |  |  |  |  |  |  |  |  |  |  |
|  | 2013 | 28 | 12.6% | 89 | 40.1% | 1 | 0.5% | 93 | 41.9% | 11 | 5.0% |
|  | 2014 | 70 | 31.8% | 76 | 34.5% | 2 | 0.9% | 57 | 25.9% | 15 | 6.8% |
|  | 2015 | 68 | 20.7% | 127 | 38.7% | 3 | 0.9% | 119 | 36.3% | 11 | 3.4% |
|  | 2016 | 53 | 16.0% | 108 | 32.5% | 3 | 0.9% | 159 | 47.9% | 9 | 2.7% |
| SLO |  | n/a |  | n/a |  | n/a |  | n/a |  | n/a |  |
| POL |  |  |  |  |  |  |  |  |  |  |  |
|  | 2013 | 8 | 23.5% | 6 | 17.6% | 0 | 0.0% | 18 | 52.9% | 2 | 5.9% |
|  | 2014 | 11 | 23.9% | 14 | 30.4% | 3 | 6.5% | 15 | 32.6% | 3 | 6.5% |
|  | 2015 | 19 | 27.9% | 16 | 23.5% | 4 | 5.9% | 29 | 42.6% | 0 | 0.0% |
|  | 2016 | 18 | 31.6% | 13 | 22.8% | 2 | 3.5% | 22 | 38.6% | 2 | 3.5% |
| NOR |  |  |  |  |  |  |  |  |  |  |  |
|  | 2013 | 102 | 48.6% | 49 | 23.3% | 0 | 0.0% | 45 | 21.4% | 14 | 6.7% |
|  | 2014 | 108 | 51.2% | 54 | 25.6% | 0 | 0.0% | 43 | 20.4% | 6 | 2.8% |
|  | 2015 | 113 | 44.3% | 56 | 22.0% | 0 | 0.0% | 72 | 28.2% | 14 | 5.5% |
|  | 2016 | 105 | 48.2% | 42 | 19.3% | 1 | 0.5% | 66 | 30.3% | 4 | 1.8% |
| FLA |  |  |  |  |  |  |  |  |  |  |  |
|  | 2013 | 37 | 39.4% | 29 | 30.9% | 0 | 0.0% | 28 | 29.8% | 0 | 0.0% |
|  | 2014 | 53 | 44.5% | 34 | 28.6% | 0 | 0.0% | 32 | 26.9% | 0 | 0.0% |
|  | 2015 | 49 | 48.0% | 29 | 28.4% | 0 | 0.0% | 24 | 23.5% | 0 | 0.0% |
|  | 2016 | 64 | 50.0% | 30 | 23.4% | 0 | 0.0% | 34 | 26.6% | 0 | 0.0% |
|  | Social and economic geography | | | | | | | | | | |
| CZE |  |  |  |  |  |  |  |  |  |  |  |
|  | 2013 | 0 | 0.0% | 2 | 40.0% | 0 | 0.0% | 3 | 60.0% | 0 | 0.0% |
|  | 2014 | 2 | 20.0% | 3 | 30.0% | 0 | 0.0% | 3 | 30.0% | 2 | 20.0% |
|  | 2015 | 2 | 12.5% | 5 | 31.3% | 0 | 0.0% | 9 | 56.3% | 0 | 0.0% |
|  | 2016 | 3 | 17.6% | 4 | 23.5% | 0 | 0.0% | 9 | 52.9% | 1 | 5.9% |
| SLO |  | n/a |  | n/a |  | n/a |  | n/a |  | n/a |  |
| POL |  | n/a |  | n/a |  | n/a |  | n/a |  | n/a |  |
| NOR |  |  |  |  |  |  |  |  |  |  |  |
|  | 2013 | 141 | 59.5% | 61 | 25.7% | 0 | 0.0% | 34 | 14.3% | 1 | 0.4% |
|  | 2014 | 153 | 62.2% | 63 | 25.6% | 0 | 0.0% | 28 | 11.4% | 2 | 0.8% |
|  | 2015 | 136 | 59.9% | 49 | 21.6% | 0 | 0.0% | 36 | 15.9% | 6 | 2.6% |
|  | 2016 | 174 | 64.9% | 49 | 18.3% | 2 | 0.7% | 43 | 16.0% | 0 | 0.0% |
| FLA |  |  |  |  |  |  |  |  |  |  |  |
|  | 2013 | 89 | 54.3% | 42 | 25.6% | 1 | 0.6% | 30 | 18.3% | 2 | 1.2% |
|  | 2014 | 88 | 49.4% | 52 | 29.2% | 0 | 0.0% | 37 | 20.8% | 1 | 0.6% |
|  | 2015 | 82 | 55.4% | 36 | 24.3% | 0 | 0.0% | 30 | 20.3% | 0 | 0.0% |
|  | 2016 | 128 | 65.6% | 38 | 19.5% | 1 | 0.5% | 23 | 11.8% | 5 | 2.6% |
|  | Media and communications | | | | | | | | | | |
| CZE |  |  |  |  |  |  |  |  |  |  |  |
|  | 2013 | 8 | 40.0% | 2 | 10.0% | 1 | 5.0% | 6 | 30.0% | 3 | 15.0% |
|  | 2014 | 5 | 20.0% | 11 | 44.0% | 1 | 4.0% | 8 | 32.0% | 0 | 0.0% |
|  | 2015 | 4 | 15.4% | 5 | 19.2% | 1 | 3.8% | 16 | 61.5% | 0 | 0.0% |
|  | 2016 | 2 | 12.5% | 6 | 37.5% | 2 | 12.5% | 5 | 31.3% | 1 | 6.3% |
| SLO |  | n/a |  | n/a |  | n/a |  | n/a |  | n/a |  |
| POL |  |  |  |  |  |  |  |  |  |  |  |
|  | 2013 | 1 | 11.1% | 1 | 11.1% | 1 | 11.1% | 6 | 66.7% | 0 | 0.0% |
|  | 2014 | 1 | 7.1% | 6 | 42.9% | 1 | 7.1% | 5 | 35.7% | 1 | 7.1% |
|  | 2015 | 5 | 29.4% | 4 | 23.5% | 0 | 0.0% | 7 | 41.2% | 1 | 5.9% |
|  | 2016 | 1 | 10.0% | 2 | 20.0% | 1 | 10.0% | 6 | 60.0% | 0 | 0.0% |
| NOR |  |  |  |  |  |  |  |  |  |  |  |
|  | 2013 | 16 | 25.8% | 15 | 24.2% | 3 | 4.8% | 27 | 43.5% | 1 | 1.6% |
|  | 2014 | 19 | 32.8% | 7 | 12.1% | 0 | 0.0% | 26 | 44.8% | 6 | 10.3% |
|  | 2015 | 17 | 27.4% | 7 | 11.3% | 0 | 0.0% | 27 | 43.5% | 11 | 17.7% |
|  | 2016 | 21 | 29.2% | 13 | 18.1% | 0 | 0.0% | 37 | 51.4% | 1 | 1.4% |
| FLA |  |  |  |  |  |  |  |  |  |  |  |
|  | 2013 | 30 | 39.0% | 23 | 29.9% | 0 | 0.0% | 19 | 24.7% | 5 | 6.5% |
|  | 2014 | 38 | 41.8% | 36 | 39.6% | 0 | 0.0% | 16 | 17.6% | 1 | 1.1% |
|  | 2015 | 59 | 56.7% | 22 | 21.2% | 4 | 3.8% | 19 | 18.3% | 0 | 0.0% |
|  | 2016 | 53 | 57.6% | 23 | 25.0% | 1 | 1.1% | 15 | 16.3% | 0 | 0.0% |
|  | Other social sciences | | | | | | | | | | |
| CZE |  |  |  |  |  |  |  |  |  |  |  |
|  | 2013 | 5 | 18.5% | 9 | 33.3% | 0 | 0.0% | 11 | 40.7% | 2 | 7.4% |
|  | 2014 | 19 | 29.7% | 18 | 28.1% | 0 | 0.0% | 25 | 39.1% | 2 | 3.1% |
|  | 2015 | 16 | 19.5% | 24 | 29.3% | 0 | 0.0% | 42 | 51.2% | 0 | 0.0% |
|  | 2016 | 25 | 29.8% | 25 | 29.8% | 0 | 0.0% | 32 | 38.1% | 2 | 2.4% |
| SLO |  |  |  |  |  |  |  |  |  |  |  |
|  | 2014 | 14 | 18.7% | 35 | 46.7% | 1 | 1.3% | 18 | 24.0% | 7 | 9.3% |
|  | 2015 | 16 | 21.6% | 26 | 35.1% | 1 | 1.4% | 31 | 41.9% | 0 | 0.0% |
|  | 2016 | 29 | 19.3% | 51 | 34.0% | 3 | 2.0% | 67 | 44.7% | 0 | 0.0% |
| POL |  |  |  |  |  |  |  |  |  |  |  |
|  | 2013 | 17 | 23.9% | 36 | 50.7% | 1 | 1.4% | 15 | 21.1% | 2 | 2.8% |
|  | 2014 | 18 | 39.1% | 18 | 39.1% | 0 | 0.0% | 10 | 21.7% | 0 | 0.0% |
|  | 2015 | 53 | 55.8% | 26 | 27.4% | 0 | 0.0% | 14 | 14.7% | 2 | 2.1% |
|  | 2016 | 17 | 33.3% | 16 | 31.4% | 0 | 0.0% | 12 | 23.5% | 6 | 11.8% |
| NOR |  |  |  |  |  |  |  |  |  |  |  |
|  | 2013 | 63 | 29.9% | 53 | 25.1% | 1 | 0.5% | 66 | 31.3% | 28 | 13.3% |
|  | 2014 | 80 | 38.8% | 42 | 20.4% | 3 | 1.5% | 69 | 33.5% | 12 | 5.8% |
|  | 2015 | 76 | 29.3% | 48 | 18.5% |  | 0.0% | 105 | 40.5% | 30 | 11.6% |
|  | 2016 | 80 | 29.2% | 62 | 22.6% | 2 | 0.7% | 113 | 41.2% | 17 | 6.2% |
| FLA |  |  |  |  |  |  |  |  |  |  |  |
|  | 2013 | 5 | 23.8% | 4 | 19.0% | 2 | 9.5% | 10 | 47.6% | 0 | 0.0% |
|  | 2014 | 7 | 30.4% | 2 | 8.7% | 1 | 4.3% | 13 | 56.5% | 0 | 0.0% |
|  | 2015 | 5 | 22.7% | 1 | 4.5% | 2 | 9.1% | 12 | 54.5% | 2 | 9.1% |
|  | 2016 | 10 | 28.6% | 10 | 28.6% | 3 | 8.6% | 12 | 34.3% | 0 | 0.0% |
|  | History and archaeology | | | | | | | | | | |
| CZE |  |  |  |  |  |  |  |  |  |  |  |
|  | 2013 | 26 | 19.4% | 21 | 15.7% | 53 | 39.6% | 27 | 20.1% | 7 | 5.2% |
|  | 2014 | 27 | 20.3% | 15 | 11.3% | 66 | 49.6% | 23 | 17.3% | 2 | 1.5% |
|  | 2015 | 29 | 21.5% | 15 | 11.1% | 70 | 51.9% | 20 | 14.8% | 1 | 0.7% |
|  | 2016 | 44 | 28.6% | 21 | 13.6% | 52 | 33.8% | 34 | 22.1% | 3 | 1.9% |
| SLO |  |  |  |  |  |  |  |  |  |  |  |
|  | 2014 | 1 | 3.8% | 0 | 0.0% | 8 | 30.8% | 17 | 65.4% | 0 | 0.0% |
|  | 2015 | 1 | 3.6% | 1 | 3.6% | 9 | 32.1% | 17 | 60.7% | 0 | 0.0% |
|  | 2016 | 2 | 6.5% | 0 | 0.0% | 7 | 22.6% | 22 | 71.0% | 0 | 0.0% |
| POL |  |  |  |  |  |  |  |  |  |  |  |
|  | 2013 | 26 | 12.4% | 8 | 3.8% | 41 | 19.6% | 130 | 62.2% | 4 | 1.9% |
|  | 2014 | 39 | 25.0% | 11 | 7.1% | 26 | 16.7% | 73 | 46.8% | 7 | 4.5% |
|  | 2015 | 29 | 19.7% | 18 | 12.2% | 40 | 27.2% | 60 | 40.8% | 0 | 0.0% |
|  | 2016 | 32 | 23.7% | 19 | 14.1% | 42 | 31.1% | 41 | 30.4% | 1 | 0.7% |
| NOR |  |  |  |  |  |  |  |  |  |  |  |
|  | 2013 | 22 | 23.9% | 4 | 4.3% | 57 | 62.0% | 5 | 5.4% | 4 | 4.3% |
|  | 2014 | 27 | 30.3% | 8 | 9.0% | 39 | 43.8% | 11 | 12.4% | 4 | 4.5% |
|  | 2015 | 20 | 18.0% | 13 | 11.7% | 45 | 40.5% | 17 | 15.3% | 16 | 14.4% |
|  | 2016 | 16 | 15.7% | 19 | 18.6% | 47 | 46.1% | 18 | 17.6% | 2 | 2.0% |
| FLA |  |  |  |  |  |  |  |  |  |  |  |
|  | 2013 | 31 | 22.0% | 29 | 20.6% | 66 | 46.8% | 11 | 7.8% | 4 | 2.8% |
|  | 2014 | 30 | 22.1% | 30 | 22.1% | 69 | 50.7% | 7 | 5.1% | 0 | 0.0% |
|  | 2015 | 30 | 31.6% | 23 | 24.2% | 34 | 35.8% | 7 | 7.4% | 1 | 1.1% |
|  | 2016 | 32 | 19.5% | 38 | 23.2% | 62 | 37.8% | 32 | 19.5% | 0 | 0.0% |
|  | Languages and literature | | | | | | | | | | |
| CZE |  |  |  |  |  |  |  |  |  |  |  |
|  | 2013 | 3 | 3.0% | 21 | 21.2% | 45 | 45.5% | 26 | 26.3% | 4 | 4.0% |
|  | 2014 | 6 | 5.7% | 18 | 17.0% | 54 | 50.9% | 27 | 25.5% | 1 | 0.9% |
|  | 2015 | 14 | 12.3% | 10 | 8.8% | 52 | 45.6% | 38 | 33.3% | 0 | 0.0% |
|  | 2016 | 4 | 3.0% | 23 | 17.4% | 51 | 38.6% | 53 | 40.2% | 1 | 0.8% |
| SLO |  | n/a |  | n/a |  | n/a |  | n/a |  | n/a |  |
| POL |  |  |  |  |  |  |  |  |  |  |  |
|  | 2013 | 24 | 7.0% | 37 | 10.8% | 135 | 39.5% | 134 | 39.2% | 12 | 3.5% |
|  | 2014 | 24 | 6.6% | 43 | 11.9% | 138 | 38.2% | 150 | 41.6% | 6 | 1.7% |
|  | 2015 | 39 | 8.5% | 46 | 10.0% | 143 | 31.1% | 230 | 50.0% | 2 | 0.4% |
|  | 2016 | 43 | 12.1% | 41 | 11.5% | 112 | 31.5% | 157 | 44.2% | 2 | 0.6% |
| NOR |  |  |  |  |  |  |  |  |  |  |  |
|  | 2013 | 23 | 16.7% | 30 | 21.7% | 47 | 34.1% | 33 | 23.9% | 5 | 3.6% |
|  | 2014 | 32 | 23.5% | 25 | 18.4% | 42 | 30.9% | 32 | 23.5% | 5 | 3.7% |
|  | 2015 | 44 | 29.5% | 23 | 15.4% | 46 | 30.9% | 31 | 20.8% | 5 | 3.4% |
|  | 2016 | 43 | 25.1% | 35 | 20.5% | 58 | 33.9% | 35 | 20.5% | 0 | 0.0% |
| FLA |  |  |  |  |  |  |  |  |  |  |  |
|  | 2013 | 37 | 14.1% | 50 | 19.0% | 150 | 57.0% | 24 | 9.1% | 2 | 0.8% |
|  | 2014 | 52 | 21.1% | 38 | 15.4% | 129 | 52.4% | 27 | 11.0% | 0 | 0.0% |
|  | 2015 | 43 | 20.2% | 33 | 15.5% | 83 | 39.0% | 53 | 24.9% | 1 | 0.5% |
|  | 2016 | 47 | 18.6% | 53 | 20.9% | 106 | 41.9% | 47 | 18.6% | 0 | 0.0% |
|  | Philosophy, ethics and religion | | | | | | | | | | |
| CZE |  |  |  |  |  |  |  |  |  |  |  |
|  | 2013 | 9 | 5.9% | 4 | 2.6% | 117 | 76.5% | 23 | 15.0% | 0 | 0.0% |
|  | 2014 | 10 | 5.1% | 6 | 3.0% | 153 | 77.3% | 29 | 14.6% | 0 | 0.0% |
|  | 2015 | 4 | 2.4% | 8 | 4.7% | 120 | 71.0% | 37 | 21.9% | 0 | 0.0% |
|  | 2016 | 13 | 7.9% | 17 | 10.3% | 107 | 64.8% | 28 | 17.0% | 0 | 0.0% |
| SLO |  | n/a |  | n/a |  | n/a |  | n/a |  | n/a |  |
| POL |  |  |  |  |  |  |  |  |  |  |  |
|  | 2013 | 15 | 10.7% | 16 | 11.4% | 51 | 36.4% | 55 | 39.3% | 3 | 2.1% |
|  | 2014 | 10 | 7.0% | 28 | 19.6% | 38 | 26.6% | 65 | 45.5% | 2 | 1.4% |
|  | 2015 | 29 | 17.9% | 14 | 8.6% | 40 | 24.7% | 78 | 48.1% | 1 | 0.6% |
|  | 2016 | 24 | 13.0% | 31 | 16.8% | 43 | 23.4% | 61 | 33.2% | 25 | 13.6% |
| NOR |  |  |  |  |  |  |  |  |  |  |  |
|  | 2013 | 9 | 8.8% | 16 | 15.7% | 43 | 42.2% | 23 | 22.5% | 11 | 10.8% |
|  | 2014 | 6 | 5.6% | 16 | 15.0% | 50 | 46.7% | 29 | 27.1% | 6 | 5.6% |
|  | 2015 | 9 | 6.6% | 20 | 14.6% | 51 | 37.2% | 35 | 25.5% | 22 | 16.1% |
|  | 2016 | 15 | 13.4% | 13 | 11.6% | 51 | 45.5% | 31 | 27.7% | 2 | 1.8% |
| FLA |  |  |  |  |  |  |  |  |  |  |  |
|  | 2013 | 11 | 7.9% | 5 | 3.6% | 83 | 59.7% | 35 | 25.2% | 5 | 3.6% |
|  | 2014 | 17 | 10.6% | 13 | 8.1% | 91 | 56.5% | 40 | 24.8% | 0 | 0.0% |
|  | 2015 | 17 | 14.5% | 8 | 6.8% | 56 | 47.9% | 34 | 29.1% | 2 | 1.7% |
|  | 2016 | 11 | 8.1% | 16 | 11.8% | 79 | 58.1% | 28 | 20.6% | 2 | 1.5% |
|  | Arts |  |  |  |  |  |  |  |  |  |  |
| CZE |  |  |  |  |  |  |  |  |  |  |  |
|  | 2013 | 1 | 1.3% | 7 | 8.9% | 43 | 54.4% | 27 | 34.2% | 1 | 1.3% |
|  | 2014 | 11 | 12.0% | 2 | 2.2% | 51 | 55.4% | 22 | 23.9% | 6 | 6.5% |
|  | 2015 | 22 | 19.6% | 7 | 6.3% | 52 | 46.4% | 31 | 27.7% | 0 | 0.0% |
|  | 2016 | 11 | 10.9% | 8 | 7.9% | 49 | 48.5% | 33 | 32.7% | 0 | 0.0% |
| SLO |  |  |  |  |  |  |  |  |  |  |  |
|  | 2014 | 0 | 0.0% | 0 | 0.0% | 1 | 100% | 0 | 0.0% | 0 | 0.0% |
|  | 2015 | 0 | 0.0% | 0 | 0.0% | 1 | 100% | 0 | 0.0% | 0 | 0.0% |
|  | 2016 | 0 | 0.0% | 0 | 0.0% | 4 | 80.0% | 1 | 20.0% | 0 | 0.0% |
| POL |  |  |  |  |  |  |  |  |  |  |  |
|  | 2013 | 0 | 0.0% | 1 | 3.0% | 7 | 21.2% | 25 | 75.8% | 0 | 0.0% |
|  | 2014 | 3 | 5.7% | 4 | 7.5% | 18 | 34.0% | 28 | 52.8% | 0 | 0.0% |
|  | 2015 | 1 | 2.9% | 1 | 2.9% | 13 | 38.2% | 19 | 55.9% | 0 | 0.0% |
|  | 2016 | 2 | 7.1% | 2 | 7.1% | 6 | 21.4% | 18 | 64.3% | 0 | 0.0% |
| NOR |  |  |  |  |  |  |  |  |  |  |  |
|  | 2013 | 1 | 1.9% | 12 | 23.1% | 25 | 48.1% | 14 | 26.9% | 0 | 0.0% |
|  | 2014 | 3 | 4.9% | 18 | 29.5% | 21 | 34.4% | 15 | 24.6% | 4 | 6.6% |
|  | 2015 | 6 | 7.3% | 14 | 17.1% | 39 | 47.6% | 20 | 24.4% | 3 | 3.7% |
|  | 2016 | 3 | 3.8% | 20 | 25.3% | 33 | 41.8% | 23 | 29.1% | 0 | 0.0% |
| FLA |  |  |  |  |  |  |  |  |  |  |  |
|  | 2013 | 6 | 9.8% | 8 | 13.1% | 33 | 54.1% | 13 | 21.3% | 1 | 1.6% |
|  | 2014 | 8 | 14.8% | 7 | 13.0% | 22 | 40.7% | 16 | 29.6% | 1 | 1.9% |
|  | 2015 | 8 | 14.3% | 7 | 12.5% | 26 | 46.4% | 15 | 26.8% | 0 | 0.0% |
|  | 2016 | 9 | 15.0% | 5 | 8.3% | 21 | 35.0% | 25 | 41.7% | 0 | 0.0% |
|  | Other humanities | | | | | | | | | | |
| CZE |  |  |  |  |  |  |  |  |  |  |  |
|  | 2013 | 0 | 0.0% | 0 | 0.0% | 0 | 0.0% | 0 | 0.0% | 0 | 0.0% |
|  | 2014 | 0 | 0.0% | 0 | 0.0% | 0 | 0.0% | 0 | 0.0% | 0 | 0.0% |
|  | 2015 | 0 | 0.0% | 0 | 0.0% | 0 | 0.0% | 0 | 0.0% | 0 | 0.0% |
|  | 2016 | 0 | 0.0% | 0 | 0.0% | 0 | 0.0% | 1 | 100% | 0 | 0.0% |
| SLO |  |  |  |  |  |  |  |  |  |  |  |
|  | 2014 | 7 | 4.8% | 3 | 2.1% | 75 | 51.4% | 30 | 20.5% | 31 | 21.2% |
|  | 2015 | 9 | 5.7% | 9 | 5.7% | 68 | 43.3% | 71 | 45.2% | 0 | 0.0% |
|  | 2016 | 6 | 3.1% | 13 | 6.8% | 69 | 35.9% | 101 | 52.6% | 3 | 1.6% |
| POL |  |  |  |  |  |  |  |  |  |  |  |
|  | 2013 | 2 | 6.1% | 3 | 9.1% | 11 | 33.3% | 17 | 51.5% | 0 | 0.0% |
|  | 2014 | 0 | 0.0% | 4 | 12.1% | 14 | 42.4% | 11 | 33.3% | 4 | 12.1% |
|  | 2015 | 2 | 6.1% | 4 | 12.1% | 12 | 36.4% | 14 | 42.4% | 1 | 3.0% |
|  | 2016 | 1 | 3.1% | 3 | 9.4% | 11 | 34.4% | 17 | 53.1% | 0 | 0.0% |
| NOR |  |  |  |  |  |  |  |  |  |  |  |
|  | 2013 | 2 | 11.1% | 1 | 5.6% | 11 | 61.1% | 4 | 22.2% | 0 | 0.0% |
|  | 2014 | 2 | 22.2% | 0 | 0.0% | 6 | 66.7% | 1 | 11.1% | 0 | 0.0% |
|  | 2015 | 2 | 10.0% | 2 | 10.0% | 7 | 35.0% | 8 | 40.0% | 1 | 5.0% |
|  | 2016 | 8 | 28.6% | 1 | 3.6% | 9 | 32.1% | 5 | 17.9% | 5 | 17.9% |
| FLA |  |  |  |  |  |  |  |  |  |  |  |
|  | 2013 | 1 | 6.7% | 4 | 26.7% | 4 | 26.7% | 6 | 40.0% | 0 | 0.0% |
|  | 2014 | 1 | 3.8% | 7 | 26.9% | 11 | 42.3% | 7 | 26.9% | 0 | 0.0% |
|  | 2015 | 2 | 9.5% | 3 | 14.3% | 6 | 28.6% | 10 | 47.6% | 0 | 0.0% |
|  | 2016 | 2 | 5.9% | 6 | 17.6% | 6 | 17.6% | 20 | 58.8% | 0 | 0.0% |

CZE Czech Republic, SLO Slovakia, POL Poland, NOR Norway, FLA Flanders
