# Supplementary material for: Journal article publishing in the social sciences and humanities: A comparison of Web of Science coverage for five European countries
Source: PLoS One. 2021 Apr 8;16(4):e0249879. doi: 10.1371/journal.pone.0249879 (PMC8031415; doi:10.1371/journal.pone.0249879)
Supplement: Attachment — Submitted filename: PLoSOne_MichalPetr_response to reviewers.docx [file pone.0249879.s016.docx]

**Rebuttal letter**

Michal Petr
Masaryk University
Rector´s Office, Zerotinovo namesti 9, 601 077 Brno, Czech Republic

30. 9. 2020

Revised cover letter to: Petr, M et al., Journal article publishing in the social sciences and humanities: a comparison of Web of Science coverage for five European countries (formerly: Coverage of journal articles in social sciences and humanities in Web of Science and their representation in citation indexes: a comparison of five European countries)

Dear PloS One Editors,

Foremost, we would like to thank both reviewers for very useful and challenging comments. However, we were struggling with making all requested changes as these sometimes pit against each other. Whereas Reviewer #1 requested shortening the text (which we consider very useful), implementing all changes requested by Reviewer #2 would make the manuscript much longer. Some Reviewer´s #2 suggestions – however undoubtedly overall relevant – goes far beyond the intended focus of this study, changes the scope and would result in the entirely new manuscript. In this respect, we decided giving priority to reducing the text, keeping our original research questions, concept, focus and methodology.

Besides concept-changing requests, we made all requested revisions. Foremost, we reduced and reformulated lengthy Results and Discussion, as Reviewer 1 suggested. At the same time, we added a few paragraphs in the manuscript, discussing Reviewer´s 2 concerns, which we consider very useful and relevant. We believe that we found the right balance and reasonably consolidated the Results and Discussion. We also paid increased attention to improving overall English to make the text clear.

We also uploaded the version with track changes. However, this version only shows our changes in the manuscript and does not contain the changes made during proofreading. Clean version with accepted changes is the final version of revised manuscript.

Below we copied the editor´s review and both Reviewer´s comments.

Our response is marked with colour and aligned with an increased indent of the paragraph, like this text

***

Editor's comments

When submitting your revision, we need you to address these additional requirements.

1. Please ensure that your manuscript meets PLOS ONE's style requirements, including those for file naming. The PLOS ONE style templates can be found at

<https://journals.plos.org/plosone/s/file?id=wjVg/PLOSOne_formatting_sample_main_body.pdf> and <https://journals.plos.org/plosone/s/file?id=ba62/PLOSOne_formatting_sample_title_authors_affiliations.pdf>

2. Please ensure that you include a title page within your main document. We do appreciate that you have a title page document uploaded as a separate file, however, as per our author guidelines (<http://journals.plos.org/plosone/s/submission-guidelines#loc-title-page>) we do require this to be part of the manuscript file itself and not uploaded separately.

Could you therefore please include the title page into the beginning of your manuscript file itself, listing all authors and affiliations.

We included the title page into the main document as required.

3. We note that you have indicated that data from this study are available upon request. PLOS only allows data to be available upon request if there are legal or ethical restrictions on sharing data publicly. For more information on unacceptable data access restrictions, please see <http://journals.plos.org/plosone/s/data-availability#loc-unacceptable-data-access-restrictions>.

In your revised cover letter, please address the following prompts:

a) If there are ethical or legal restrictions on sharing a de-identified data set, please explain them in detail (e.g., data contain potentially sensitive information, data are owned by a third-party organization, etc.) and who has imposed them (e.g., an ethics committee). Please also provide contact information for a data access committee, ethics committee, or other institutional body to which data requests may be sent.

b) If there are no restrictions, please upload the minimal anonymized data set necessary to replicate your study findings as either Supporting Information files or to a stable, public repository and provide us with the relevant URLs, DOIs, or accession numbers. For a list of acceptable repositories, please see <http://journals.plos.org/plosone/s/data-availability#loc-recommended-repositories>.

We will update your Data Availability statement on your behalf to reflect the information you provide.

We uploaded the data in the repository.

4. Please upload a copy of Figures 10 and 11, to which you refer in your text on pages 17, 23 and 27. If any figure is no longer to be included as part of the submission please remove all reference to it within the text.

We fixed references and figures.

5. Please include a copy of Table 1 which you refer to in your text on page 19.

Table 1 was at the very beginning of the manuscript.

6. Please include captions for your Supporting Information files at the end of your manuscript, and update any in-text citations to match accordingly. Please see our Supporting Information guidelines for more information: [http://journals.plos.org/plosone/s/supporting-information](about:blank)

We included captions at the end of the manuscript and checked in-text references.

Reviewers' comments


Reviewer #1

This paper compares comprehensive national records of papers in social sciences and humanities with WoS lists of such papers. Trends over time in Flanders and Norway are compared against Poland, Czech Republic and Slovakia. The paper is solid and I have just a few questions. The text does go on at some length and should be shortened. There were pages and pages of Discussion when there really wasn’t that much to discuss, results are just being repeated multiple times.

Thank you for this suggestion. We shortened the text, mainly in the Results section. Please, see the revised manuscript.

The formatting of the figures made it impossible to consider them in the review. The figures came after the text with no captions associated with them. I don’t know whether this was journal submission requirements or author choice, either way I ended up just ignoring the figures.

Graphs were uploaded as separate files, according to PLoS One requirements. We fixed all in-text references and included graphs in the text.

On page 4 the way ESCI is described makes one wonder why the journals aren’t just included in WoS. Maybe improve explanation.

P. 5 “revealing publication patterns of the coverage and ranking of journals in international comparison”
Confusing phrasing, rework the sentence.

p. 5 “A publication in a WoS indexed journal is in most SSH disciplines still seen as a success of its kind and also as an indication of higher quality in publication profile [23]. If this is true, then the ranking of journals might play a role in this process.”

What process? And if what is true? That WoS is seen as higher quality? If it might not be true that it is seen this way, then why did you say it?

We looked at how the Introduction could be expressed more clearly, given these questions from Reviewer #1. Finally, we edited the Introduction from its start and improved the explanations.

Table 1 – numbers in tables should be right justified and ,’s should be used to demarcate 1000’s. And how are the rows in this table ordered? Largest to smallest would be a good order.

We updated all Tables as suggested and added following sentence under the Table 1: *“Disciplines are ordered as they appear in the Frascati classification scheme.”*

“share of articles” sounds like the wrong phrase. It should probably be “article share” Share of articles sounds like the % of articles that are X. You want articles as a % of X.

Thank you. With our proofreader, we rephrased “share of articles” to “article share” and improved overall English.

Table 3 Can you not spell out the country names? And where is the data for Norway?

We added the country names legend below tables. The data from Norway was present – there was a mistake in splitting the table in two pages. We fixed this.

p. 18 You need to use the field name in the first sentence or two discussing field data in every section. Do not rely on the section heading. Sometime the sentences here can read like they refer to all the data.

Concerning the very first comment on lengthy Results, we have reformulated the whole section. Namely, we deleted headings, reduced the text describing each discipline and substituted with the description of findings in publication patterns. Please, see the manuscript.

p. 23 “ this leads to the same threshold whether” - meaning unclear, rephrase

We rephrased an unclear sentence.

Reviewer #2

- Data for four (or even three) subsequent single years are insufficient to base conclusions on concerning publication dynamics, let alone trends.

National databases are heterogeneous as for the scope, coverage, structure of publication types and availability of the data. Databases are also unique from the perspective of historical and political background. From that point of view, comparing data from databases with unique data structure and working conditions is highly problematic. We decided to analyze data for the timeframe 2013–2016 because this was the timeframe for which the data was available across all databases. In some databases, data is collected retrospectively with some delay. Expanding is problematic when we need to align all countries.

In this study, the data from national databases needed were post-processed to be mutually comparable and to be linked to the information about indexing each journal in each citation index in each year of the study. This procedure is only partly doable automatically. A considerable part of the work needs to be done manually (mostly linking ISSNs and titles to WoS journal lists and verification of the coverage of journals in given years). Therefore, this timeframe is, to a certain extent balanced with great accuracy of the data and with a couple of months (sic!) of data cleaning in background.

Following reviewers recommendations, we achieved to add the year 2016 for Flanders data to align the timeframe with other countries. Slovakia has no older data in the database than 2014. Even when showing 4-years data, we can see overall trends and differences between countries at least in disciplines with a sufficient number of publications. Even in the pre-revised version of the manuscript, we did not base conclusions in disciplines with small publication base and low coverage.

We have added the following explanation in the Data and Methods section: *“National databases are unique as for the scope, coverage, structure of publication types and availability of the data. Databases have also different historical and political background. From that point of view, comparing data from databases is highly problematic. Since we endeavour to study all SSH disciplines, we accepted the limited timeframe 2013–2016 for which the data was available across all databases.”*

- The analysis of top half IFs vs. bottom half IFs is crude; Subject Category - normalized IFs would be preferable

Since quartiles are calculated on the basis of the IF ranking within each WoS Category, they already form a crude subject normalization. We use quartiles because it is an understandable way how to show the position of the journal in the WoS category. Also, in many countries, it is customary to refer to quartiles in evaluation-related procedures. Quartiles are here perceived as “quality” sign. Therefore, the idea of this article was to compare the proportions of quartile zones across countries and to show, on the one hand, if the coverage is changing and on the other hand if the perceived “quality” of journals is changing. We explained this directly in the Introduction and also in Data and Methods section. We are of the opinion that IF or any other finer-grained metric would be kind of different information and not so meaningful for the purpose of the study.

We have **rephrased the Introduction** as follows:

*“In this study, we consider IF to be an indication of a journal’s citation impact. Following this rationale, we take a journal’s quartile ranking in the JCR to be an indication of the journal’s quality requirements, selectivity, and scholarly impact. Those with higher rankings (Q1 and Q2) are more demanding journals. In this paper, we do not discuss whether IF is an appropriate indicator for evaluating research. We only use it at the journal level, which it was designed for. At the same time, we do not claim that WoS-indexed journals are better than non-WoS-indexed journals. Our selection of indicators reflects the fact that all five countries in our study make use of bibliometric indicators in their national funding and evaluation systems. This common trait in these countries represents just one part of the greater diversity of evaluation systems across Europe.”*

We have added the following part to the **Data and methods section**:

*“In many countries, it is customary to refer to quartiles in evaluation-related procedures. Quartiles are perceived as a sign of quality. Therefore, in this paper we seek to demonstrate on one hand if coverage is changing and on the other if the perceived quality of the journals in which articles are published is changing.”*

- Differences between (groups of) countries should be tested statistically where possible

Since we work with simple descriptive statistics, we do not consider statistical testing as necessary in this specific situation. It is not clear, what is a benefit of statistical testing for the present study when we show the simple article counts and shares without using more dimensions in one figure.

- The decision concerning journals classified in multiple citation indexes to include these in SCI-E and SSCI if possible, and not partly to A&HCI when relevant, might bias findings for Social Sciences as well as for Humanities (p. 8). Preferably, this should be changed, if not, this potential bias should be discussed.

For the article share and coverage the presence in multiple citation indexes does not play a role. For the representation in citation indexes we counted only one citation index with the decision strategy explained in the manuscript – priority was given to JCR indexation (with IF), then we searched in AHCI and finally in ESCI. The explanation for this decision was the point of view of national evaluation systems and the perception of journal “prestige” or “quality”.

We looked at the extent to which journals are indexed both in AHCI and SCI-E or SSCI and added the text to the Data and methods section and found that there are 4.4 % articles with multiple assignments to AHCI and SCI-E/SSCI of the total number of WoS-indexed articles. Then there are 27.6 % articles with multiple assignments to AHCI and SCI-E/SSCI out of all AHCI-indexed articles. It is a very interesting phenomenon; however, it goes beyond the extent of this study. Changing the counting method in the way the Reviewer suggests would cause enormous changes in the master dataset, including new time-consuming cleaning and completing the data. Looking at journals with multiple citation index assigned, we revealed many specific situations, deserving manual decision. Then, the deeper conceptualization is needed, what does it mean for the performance in SSH. Although the representation of these journals with multiple indexes is remarkable, we think that treating multiple indexes as separate values at the level of the journal would cause potential inaccuracy and would be of rather low added value to this particular research questions.

We added the following sentence in the Data and Methods section.

*“We also assigned a journal to the AHCI if it was present in both the AHCI and the SCI-E/SSCI but did not have an IF.”*

Then we discussed this as a limitation in the Discussion as follows:

*“Another limitation is rooted in the indexation of some journals in multiple databases, foremost those indexed both in the AHCI and the SCI-E / SSCI-E. This implies a risk of bias regarding the extent to which the SSH are covered in each individual citation index. Presence in multiple citation indexes does not affect article share and overall coverage. In terms of article representation in citation indexes, we decided to link every journal in each year with just one citation index, preferring the SCI-E/SSCI-E over the AHCI, as one of our research questions focuses on changes in the number of articles published in top-tier journals.”*

The paper offers an interesting comparison between 3 West Slavic central European countries and two non-English Germanic ones in North West Europe, concerning their journal article output in SSH. The study of SSH output has a considerable history that, unfortunately,has not been given due consideration. Not much is said about the three elephants in the room: English (the dominant language in scholarly publishing), German (an important language in central and north-western Europe, and Russian the most important Slavic scholarly language in many fields. Especially in the humanities, English is less often used than in most (social) sciences, as opposed to German and Russian.

Thank you for this suggestion. We have added the following part to the Discussion:

*“English-language journals are overrepresented in WoS. Hence, countries or disciplines publishing more in English are far more likely to be well represented in WoS. This partly explains the lower coverage of humanities publications.”*

Kulczycki et al. (2020), <https://asistdl.onlinelibrary.wiley.com/doi/full/10.1002/asi.24336>, have found that the importance of Russian and German is fairly limited in journal publishing in CEE countries nowadays, accounting for, respectively, 0.4 to 1.0% and 1.7 to 2.3% of SSH articles. In other words, both languages are historically important in some of the countries under scrutiny but no longer play a major role. For this reason, we don't specifically mention them in the article

The perhaps most interesting aspect of the paper is the difference in evaluation and reward systems ("PRFS") and in particular the way this relates to adaptive publication strategies of scholars. However, the various PRFSs are described rather summarily for a full appreciation of the findings of the study.

Thank you for this relevant suggestion. This is an aspect that we are usually intensely interested in as well. However, studying relationships between PRFS and publication strategies directly in the manuscript was not the goal of this paper. Expectedly, it would take a lot of space – especially when describing the details how publications are discounted in those models – whereas Reviewer #1 asked for reducing the text length. We edited the Introduction and added references to recently published research as follows rather than describe each PRFS in detail.

*Debackere, K., Arnold, E., Sivertsen, G., Spaapen, J., & Sturn, D. (2018). Performance-Based Funding of University Research. Publications Office of the European Union.* [*https://doi.org/10.2777/644014*](https://doi.org/10.2777/644014)*.*

*Zacharewicz, T., Lepori, B., Reale, E., & Jonkers, K. (2018). Performance-based research funding in EU Member States—A comparative assessment. Science and Public Policy.* [*https://doi.org/10.1093/scipol/scy041*](https://doi.org/10.1093/scipol/scy041)

Finally, although the importance of books for PRFSs is mentioned, the present study is not able to present much data on these. It is not made clear how well the Book Citation Indexes cover monographs and edited volumes.

We agree that books are crucial for PRFSs too. We did not provide any data on this because the main point of the paper was to study the **article** share, coverage and presence in WoS CC citation indexes with the finer-grained distinction of quartiles within JCR across all SSH disciplines in Frascati classification. It is not possible to cover all topics related to all publication patterns in SSH. The coverage of BCI is very limited in the SSH and is documented by Aksnes and Sivertsen (2019), which is also referenced in the manuscript.

Major revisions are recommended to meet the concerns raised above.

Typo's / mistakes: Table 2 should be replaced by Table s2. CZE in Table 2 should be NOR on p.11.

We fixed these mistakes.
