# Supplementary material for: Journal article publishing in the social sciences and humanities: A comparison of Web of Science coverage for five European countries
Source: PLoS One. 2021 Apr 8;16(4):e0249879. doi: 10.1371/journal.pone.0249879 (PMC8031415; doi:10.1371/journal.pone.0249879)
Supplement: Attachment — Submitted filename: Response to Reviewers.docx [file pone.0249879.s017.docx]

**Rebuttal letter 2**

Michal Petr
Masaryk University
Rector´s Office, Zerotinovo namesti 9, 601 077 Brno, Czech Republic

15. 2. 2021

Revised cover letter to: Petr, M et al., Journal article publishing in the social sciences and humanities: a comparison of Web of Science coverage for five European countries.

Dear PloS One Editors,

We are thankful for the Reviewer’s #2 comments that inspired us to make some more revisions and explain some critical points in the manuscript. We also added some extra references, addressing significant topics suggested by Reviewer’s #2. We tried to reflect the Reviewer‘s #2 concerns; however, we still feel that making all changes requested by Reviewer #2 would change the article's scope entirely. For instance, reviewer #2 asked addressing the effects of changes in PRFS on changes in publication patterns. We feel that addressing this point would make this text on the one hand too extensive in the scope and length, on the other hand too general to address these points to a deserved extent. Further, Reviewer #2 requested replacing the IF quartiles with more finer-grained impact measure. We want to remark that using IF quartiles in our article is entirely intentional as several national evaluations use this exact level for assessing performance. However, we addressed several topics in the article, provided some extra explanations and added references to document the Reviewer‘s #2 points.

Reviewer #2 finally says: „Although the revised paper deals satisfactory with a few points have been raised in the original comments, it fails to address several important points.”

In this article, we do not aim for comprehensiveness; the added value lies mainly in comparing the data from national databases from the perspective of the perception of publication performance by evaluation systems. We do not argue with the overall relevance and importance of recommendations that we did not implement. However, we still see that some implementations (mentioned above) are far beyond the paper's current scope and would significantly change it. Therefore, we prefer to focus on the topics we set up at the beginning of the research, as stated in the article.

With full respect to the experience of Reviewer #2, we would like to thank you for these beneficial recommendations, which we will be happy to address in further research projects. We kindly ask the Editor to reflect in his decision our argumentation that we carefully considered and still see as reasonable.

Below we copied the Reviewer´s #2 comments.

We also replaced the figures with PACE-fixed, as requested by the Editor.

Our response is marked with colour and aligned with an increased indent of the paragraph, like this text

Reviewers' comments


Reviewer #2: It is laudable that the authors have provided additional data for Flanders for 2016, but a four-year period is still way too short to detect a trend. It might be possible to test for differences between, for instance, early and late years, but unfortunately, the authors decline to do this. This makes the paper less suitable for PLOS ONE.

A minor point is that the paper still does not pay proper attention to earlier studies dealing with publication changes in SSH.

National bibliographic databases are relatively new data sources, and we benefit from the fact that they cover full national output. WoS has been used as the main data source in all periods of bibliometric research on publication patterns, but national databases are used nowadays as an emerging data source with a great potential to analyse publication patterns beyond WoS. However, national bibliographic databases have limitations, such as differences in comprehensiveness, data structure, scope, covered period etc. Limitations having effect e.g. in the period analyzed in our article, pin the territory where our data can be reliable in mutual comparison. Expanding this period is problematic. Using just Web of Science data would make the work several times easier and allow to extend the timeframe of the dataset easily, but we decided to strengthen the focus on referencing the relevant literature on this and on describing these differences. We added the following text and a couple of relevant references to the very beginning of the Introduction:

*From ca the 1980s, bibliometric methods became increasingly anchored in science policy and research evaluation. In this context, bibliometric research refocused from supporting sociological theories to research evaluation topics: science mapping, systemic effects of evaluation procedures, developing new indicators or studying publication patterns [1]. The common attribute of the recent era in bibliometric scholarship is using publication metadata as the primary data source. Major international bibliographic database Web of Science (WoS) has been used in research dealing with publishing and citation characteristics and patterns changes in the social sciences and humanities (SSH), as summarized by Nederhof [2] and recently by Franssen and Wouters [1]. In 2006, Archambault et al. [3] discussed the limits of WoS for any comparative analysis in terms of SSH output due to the language-related database bias. As documented by Franssen and Wouters [1], the recent establishment of comprehensive national databases has opened a new direction of bibliometric studies of SSH allowing for a complete picture of the publication practices by covering more publication types, sources, and research with local relevance than highly selective international databases WoS and Scopus do [4]. However, national databases are relatively new and limit the research by having a different scope and comprehensiveness. In this descriptive study, drawing on the latest bibliometric research approaches and results using national databases as a main data source, we aim to expand knowledge about publication patterns in journal publishing in SSH. More specifically, on an aspect of performance perceived by national evaluation systems in several countries as a sign of good performance, i.e. ranking of journals. [1]* *Franssen T, Wouters P. Science and its significant other: Representing the humanities in bibliometric scholarship. Journal of the Association for Information Science and Technology. 2019;70:1124-1137. doi: 10.1002/asi.24206.*

*[2]* *Nederhof A. Bibliometric monitoring of research performance in the Social Sciences and the Humanities: A Review. Scientometrics. 2006;66:81–100. doi: 10.1007/s11192-006-0007-2.*

*[3]* *Archambault E, Vignola‐Gagne E, Cote G, Lariviere V, Gingras Y. Benchmarking scientific output in the social sciences and humanities: The limits of existing databases. Scientometrics. 2006;68(3):329–342. doi: 10.1007/s11192-006-0115-z.*

As for the trends, we describe the developments in the results section of the manuscript but conclude with more general ideas not entirely dependent on the trends: comparison of countries, main differences and relevant background.

Concerning the use of quartiles to measure journal impact: a small increase in the IF of a journal may raise it to another quartile; worse, a journal's decrease in IF may be less than that of one or more journals originally ranked in a higher quartile, leading to its promotion to a higher quartile. Using more fine-grained impact measures helps to detect and prevent such statistical problems. This point has not been addressed properly in the revised paper.

Although we fully agree with the above-mentioned behaviour of the indicators, we would like to point out that it was not the paper's aim to evaluate journals, assess disciplines, or build a comprehensive picture of SSH. We conducted a descriptive analysis of the state of affairs, expanding the current picture of SSH using national databases as a data source. Our goal is to detect publication patterns in the specific area that is used for evaluating research in several national evaluation systems. Therefore, dealing with small changes to IF influencing the journal’s quartile is not entirely relevant if researchers perceive Q1 journals to be somehow special. For instance, in the Czech Republic, the evaluation methodology ranks journals in IF quartiles strictly due to journals´ IF in the year of publication without paying attention to the influence of small differences in the IF value on the line between two quartiles. In the present article, we show the distribution of journals in citation indexes and more specifically in IF quartiles in the way the national systems use quartiles for evaluating research. We translated this logic with all its imperfections of approximation methods of the national evaluation. We believe that we explained this intention satisfactory in the manuscript.

The paper's choice to not include journals with an IF partly to AHCI when these are both indexed in AHCI, and other citation indexes considerably limits the paper's relevance for the humanities, as does, possibly to a lesser extent, the exclusion of BCI items.

The paper's main aim is to analyze the coverage of articles, which is clearly stated in the title and the manuscript. The decision not to duplicate items in each country´s dataset is, as we believe, suitable for the aim we established (see also above). We consider the decision strategy in deduplication of more citation indexes assigned to one journal appropriate to determine the discipline's performance aligned to the method of evaluation systems. Including journals partly in AHCI when these are also indexed in JCR is also possible and relevant for some cases. Nevertheless, we believe that humanities are not reduced in the results in any manner, as we show these articles preferably in the JCR-indexed category in accordance with the assumption that this category performs better in the evaluation system. Also, for the comparison of different countries from the point of view of the presence of articles in JCR-indexed journals, showing these duplicates in AHCI does not play a role

Regrettably, the effect of changes in PRFS (reward and evaluation systems) on changes in publication patterns is still not more fully addressed. This distracts from the potential value of the paper.

We agree that analysis of the effects of PRFS’s is an important topic deserving of extensive discussion but argue that including such an analysis would need – a lot of explanation and would make the article lengthy. Instead of this, we added several works (references) in the Discussion (p. 27) that have earlier studied the PRFS’s effects (and demonstrated how complicated it is to trace such effects), some of which also brought some general discussion of this:

Butler L. What happens when funding is linked to publication counts? In: Moed H, Glänzel W and Schmoch U (eds). Handbook of Quantitative Science and Technology Research; 2005. pp. 389–405.

Butler L. Explaining Australia’s Increased Share of ISI Publications–the Effects of a Funding Formula Based on Publication Counts. Research Policy. 2003;32(1):143–55. doi: 10.1016/S0048-7333(02)00007-0.

Aagaard K, Bloch C, Schneider JW. Impacts of Performance-Based Research Funding Systems: The Case of the Norwegian Publication Indicator. Research Evaluation. 2015; 24(2):106–117. doi: 10.1093/reseval/rvv003.

Moed HF. UK Research Assessment Exercises: Informed Judgments on Research Quality or Quantity? Scientometrics. 2008;74(1): 153–161. doi: 10.1007/s11192-008-0108-1.

Hammarfelt B, De Rijcke S. Accountability in Context: Effects of Research Evaluation Systems on Publication Practices, Disciplinary Norms and Individual Working Routines in the Faculty of Arts at Uppsala University. Research Evaluation. 2015;24(1):63–77. doi: 10.1093/reseval/rvu029.

Although the revised paper deals satisfactory with a few points have been raised in the original comments, it fails to address several important points.

We do not aim for comprehensiveness; the added value lies mainly in comparing the data from national databases from the perspective of the perception of publication performance by evaluation systems. We do not argue with the overall relevance and importance of recommendations that we did not implement. However, we still see that some implementations (e.g. effect of changes in PRFS) are far beyond the paper's current scope and would significantly change it. We feel that addressing several recommended points would make this text on the one hand too extensive in the scope and length, on the other hand too general to address these points to a deserved extent. Therefore, we prefer to focus on the topics we set up at the beginning of the research, as stated in the article.

With full respect to the experience of Reviewer #2, we would like to thank you for these very useful recommendations, which we will be happy to address in further research projects.
